# Stiffness increases with myofibroblast content and collagen density in mesenchymal high grade serous ovarian cancer

**DOI:** 10.1038/s41598-021-83685-0

**Published:** 2021-02-18

**Authors:** Virginie Mieulet, Camille Garnier, Yann Kieffer, Thomas Guilbert, Fariba Nemati, Elisabetta Marangoni, Gilles Renault, Foucauld Chamming’s, Anne Vincent-Salomon, Fatima Mechta-Grigoriou

**Affiliations:** 1 Stress and Cancer Laboratory, Institut Curie, PSL Research University, Equipe labelisée Ligue Nationale Contre le Cancer, 26, rue d’Ulm, 75248 Paris Cedex 05, France; 2 Inserm, U830, 75248 Paris, France; 3 IMAG’IC Facility, Institut Cochin, INSERM U1016, CNRS UMR 8104, Université de Paris UMR-S1016, 75014 Paris, France; 4 Laboratory of Preclinical Investigation, Institut Curie, 26, rue d’Ulm, 75248 Paris Cedex 05, France; 5 Life Imaging Facility, University of Paris, “Imageries du Vivant” (PIV) Platform, Institut Cochin, 22 rue Mechain, 75014 Paris, France; 6 Assistance publique-hôpitaux de Paris, HU Paris Ouest Site Georges Pompidou, 20 rue Leblanc, 75015 Paris, France; 7 Department of Diagnostic and Theranostic Medicine, Institut Curie Hospital Group, 26, rue d’Ulm, 75248 Paris Cedex 05, France

**Keywords:** Cancer, Gynaecological cancer, Ovarian cancer

## Abstract

Women diagnosed with high-grade serous ovarian cancers (HGSOC) are still likely to exhibit a bad prognosis, particularly when suffering from HGSOC of the Mesenchymal molecular subtype (50% cases). These tumors show a desmoplastic reaction with accumulation of extracellular matrix proteins and high content of cancer-associated fibroblasts. Using patient-derived xenograft mouse models of Mesenchymal and Non-Mesenchymal HGSOC, we show here that HGSOC exhibit distinct stiffness depending on their molecular subtype. Indeed, tumor stiffness strongly correlates with tumor growth in Mesenchymal HGSOC, while Non-Mesenchymal tumors remain soft. Moreover, we observe that tumor stiffening is associated with high stromal content, collagen network remodeling, and MAPK/MEK pathway activation. Furthermore, tumor stiffness accompanies a glycolytic metabolic switch in the epithelial compartment, as expected based on Warburg’s effect, but also in stromal cells. This effect is restricted to the central part of stiff Mesenchymal tumors. Indeed, stiff Mesenchymal tumors remain softer at the periphery than at the core, with stromal cells secreting high levels of collagens and showing an OXPHOS metabolism. Thus, our study suggests that tumor stiffness could be at the crossroad of three major processes, i.e. matrix remodeling, MEK activation and stromal metabolic switch that might explain at least in part Mesenchymal HGSOC aggressiveness.

## Introduction

Epithelial ovarian cancers are among the most aggressive gynecological tumors, making them the fifth cause of cancer death in women in western countries. Their aggressiveness is mainly due to the silent evolution of the disease in the peritoneal cavity until advanced stages and is commonly associated with poor prognosis. To date, standard treatments consist of the combination of surgery and chemotherapy, a full tumor resection remaining one of the main prognostic criteria for patient survival. However, even if the patients usually respond to the first round of chemotherapy, many of them relapse, acquire resistance and ultimately die from the disease. Up to recent years, ovarian cancers have been classified regarding their histological subtype, grade and stage, high-grade serous ovarian cancers (HGSOC) representing the vast majority (75%) of total ovarian cancers. Despite the development of PARP inhibitors^1–15^ or antiangiogenic drugs^6,7,16–26^, beneficial therapeutic strategies still need to emerge. Recently, efforts have been engaged to better stratify HGSOC and decipher the molecular mechanisms that govern tumor progression. Based on multi-omics data, including genomic, transcriptomic, proteomic and metabolomic, different studies have highlighted the existence of distinct molecular entities of HGSOC^27–42^. Interestingly, the “Fibrosis” or “Mesenchymal” HGSOC molecular subtype has been identified in all studies and is systematically associated with poor patient survival, thus representing an unmet medical need. Our laboratory uncovered one of the first molecular mechanisms, which depends on the miR-200 family of microRNA^29,31,43,44^. Indeed, by combining transcriptomic data with miR-141/200a expression level, we identified transcriptomic signatures that differentiate Mesenchymal versus Non-Mesenchymal HGSOC. Importantly, this signature is mainly composed of stromal genes encoding extracellular matrix (ECM) proteins and ECM remodeling enzymes^29,31,43^. Moreover, Mesenchymal tumors are characterized by a high content of Cancer-Associated Fibroblasts and accumulation of ECM proteins, such as collagen and fibronectin^27–29,44–50^.

In this context, we considered that the stroma could be a key actor of Mesenchymal HGSOC aggressiveness, and we thus addressed the role of stiffness in this specific molecular subtype of ovarian cancer. There is an increasing number of studies conducted in various cancers that have recently implicated tumor stiffness in tumor progression and response to treatment^51–56^. Indeed, benign tumors have been shown to be softer than malignant ones^57,58^. Interestingly, one of the major causes of tumor stiffness is related to the increased deposition of ECM in the tumor microenvironment. ECM-dependent mechanobiology in tumors is highly complex and involves matrix cross-linking, force-mediated matrix remodeling, osmotic pressure due to blood and lymphatic vessels leakage, solid pressure and jamming (decreased cell movement) mediated by cancer cell growth^59^. Indeed, collagen cross-linking often promotes tissue stiffening during transformation^60^. Moreover, stiff substrates are sufficient to transform healthy cells into their malignant counterparts in vitro^61^. In addition, matrix stiffening enhances both cancer and stromal cells migration and invasion^62^. Finally, in a patient-derived xenograft (PDX) mouse model of luminal B breast cancer, tumor stiffness increases during tumor development and is associated with a fibrotic reaction^63^. However, in ovarian cancer, studies investigating the impact of ECM stiffness on tumor progression and chemoresistance gave discrepant results^64^. Indeed, an inverse correlation has been established between stiffness and metastatic potential by studying ovarian cancer cell lines and cancer cells isolated from patient ascites^65,66^. Moreover, ovarian cancer cell lines were more proliferative and chemo-resistant on soft substrates, a process involving the Rho-ROCK pathway^67^. These unexpected results highlighted the importance of studying the impact of stiffness on cancer cell behaviors in 3-dimensions (3D). Indeed, an enhanced proliferation and chemoresistance was observed when ovarian cancer cell lines were cultured on stiff 3D bioengineered peptide-functionalized hydrogels^68^. Lastly, tissue mechanics also controls cancer cell metabolism to sustain tumor growth^69^. Indeed, in breast cancer, tumor stiffening induces a metabolic switch in cancer and stromal cells to support malignancy^70^.

Based on these observations, we hypothesized that the desmoplastic reaction occurring in Mesenchymal HGSOC could modulate tumor mechanical properties and in turn enhance tumor progression through ECM remodeling and mechano-transduction pathway regulation. To address that question, we took advantage of Patient-Derived Xenografts (PDX) mouse models engrafted with HGSOC tumors isolated from patients. Although more difficult to establish than mouse models with injected cancer cell lines, these engrafted patient tumors have been shown to fully recapitulate HGSOC histological and molecular features^34,41,71–73^. We studied 3 distinct PDX models either derived from human Mesenchymal or Non-Mesenchymal HGSOC subtypes. We first observed that tumor stiffness, measured by supersonic shear wave elastography (SWE) in vivo, strongly correlates with tumor growth in Mesenchymal HGSOC, while Non-Mesenchymal HGSOC mainly remain soft as they grow, thereby demonstrating that tumor stiffening is specifically associated with the Mesenchymal HGSOC molecular subtype. In line with this, we uncovered that tumor stiffening is linked to high myofibroblast content, accumulation of ECM proteins and contractile collagen network. In addition, we observed that the MAPK/MEK (Mitogen-activated protein kinase/Mitogen-activated ERK protein kinase kinase) pathway is activated in Mesenchymal HGSOC upon stiffening. Finally, we took advantage of PDX models, in which human stroma is replaced by the mouse one, to define the respective stiffness-dependent transcriptomic signatures of epithelial and stromal compartments using species-specific microarrays. We observed that tumor stiffening is associated with glycolytic signature in cancer cells, together with a switch from an oxidative phosphorylation (OXPHOS) to a glycolytic metabolism in stromal cells. Thus, our work reveals that tumor stiffness could be the link between several major events occurring upon Mesenchymal HGSOC growth, such as matrix accumulation and remodeling, MEK activation and stromal metabolic switch.

## Results

### Tumor stiffness increases with growth of Mesenchymal HGSOC

**Figure 1:**
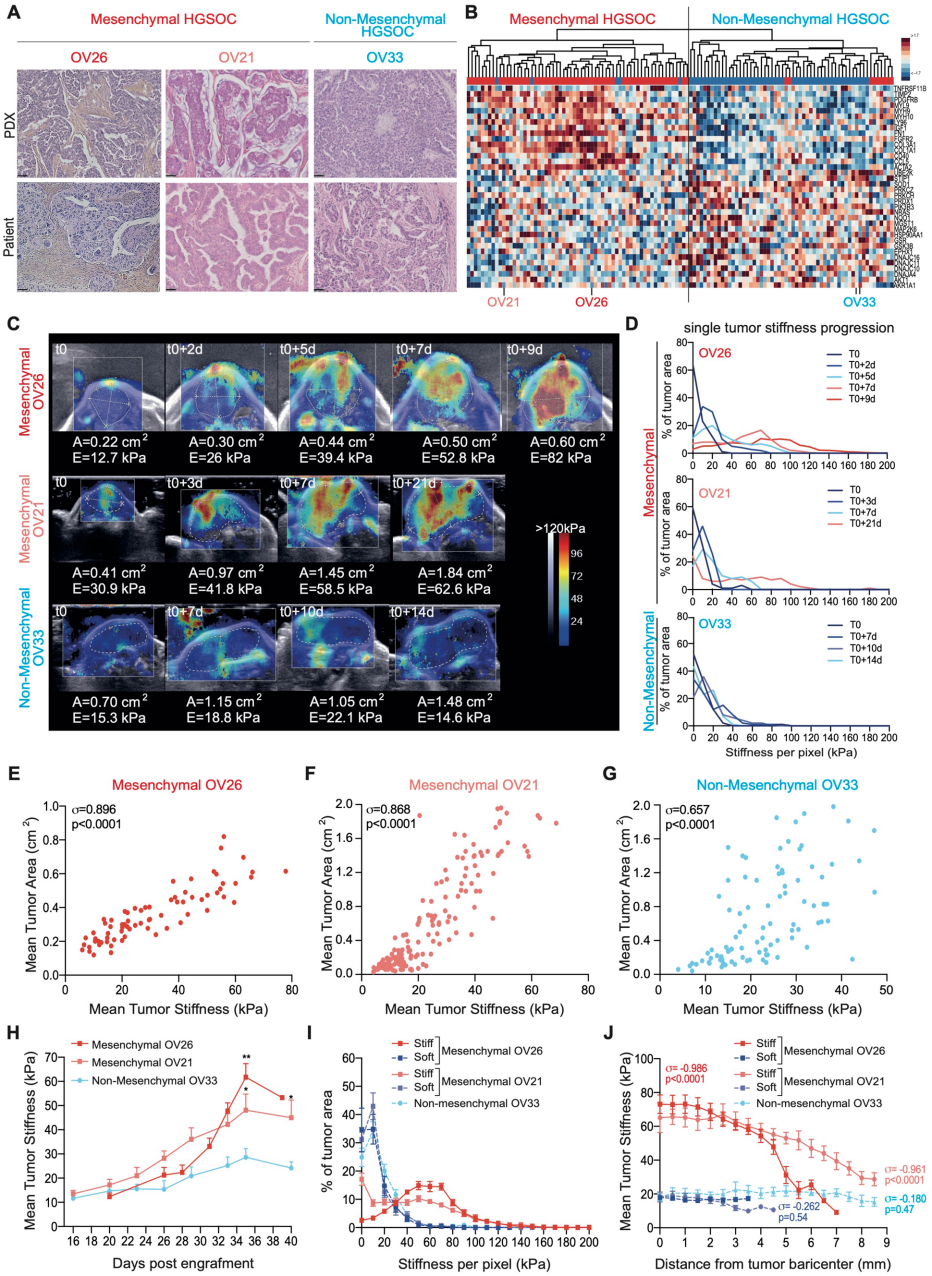
Tumor stiffness increases with growth of mesenchymal HGSOC. (**A**) Representative views of HES staining from PDX mouse models (Up) and corresponding human HGSOC (Bottom). Mesenchymal (OV26, OV21) and Non-Mesenchymal (OV33) PDX are shown. Scale bar, 50 μm. (**B**) Hierarchical clustering using Euclidean distance and Complete agglomeration method based on Mesenchymal and Non-Mesenchymal gene signatures (defined in^29^) from Institut Curie’s HGSOC data set. Each row represents a gene, and each column a tumor, with 107 HGSOC patients and 13 PDX. OV26, OV21 and OV33 are indicated. Blue and red squares indicate gene expression in each tumor below and above the mean, respectively. Color saturation indicates magnitude of deviation from the mean. The dendogram of samples (above the matrix) allows classification of patient and PDX in Mesenchymal (red) and Non-Mesenchymal (blue) subgroups. (**C**) Representative colored stiffness maps in the transverse plan of Mesenchymal and Non-Mesenchymal PDX tumor growth over time. Colored map represents the Young’s modulus value (**E**) for each pixel, stiffness scale ranging from 0 kPa (blue) to 120 kPa (red). Dotted lines, drawn by hand, delineate tumor border and area. t0 corresponds to the first day of tumor stiffness measurement, and the following days of measures are indicated. (**A**) Stands for tumor area and (**E**) for mean tumor stiffness per pixel at each time point. (**D**) Variations of stiffness values in tumor area over time. The total tumor area occupied by pixels of a specific stiffness value (pixel stiffness range: 0 to 200 kPa), inside the same representative tumor as in (**C**), from t0 and all along measurements (d, in days) in PDX models. Data are expressed as percentages rather than in bins in order to compensate for the increasing number of pixels obtained as tumors grow. (**E**–**G**) Correlation plots between mean tumor stiffness and mean tumor area upon growth of tumors from Mesenchymal OV26 (n = 22) (**E**) and OV21 (n = 30) (**F**), and Non-Mesenchymal OV33 (n = 18) (**G**) PDX models. Each dot refers to a single tumor measurement at a given time. The number of measures per tumor (m = 73 (**E**), 156 (**F**), 91 (**G**)), depends on the PDX follow-up duration limited by ethical concerns. Correlation coefficient *σ* and *P* value are based on Spearman’s rank correlation test. (**H**) Mean tumor stiffness curves over time for Mesenchymal OV26 (n = 20) and OV21 (n = 22) (**F**), and Non-Mesenchymal OV33 (n = 16) PDX models. *P* values are based on Welch's t-test. (**I**) Histograms of stiffness values in tumor area. The total tumor area occupied by pixels of a specific stiffness value (pixel stiffness range: 0 to 200 kPa) between soft and stiff Mesenchymal OV26 (soft: dark blue dashed line, n = 8; stiff: red line, n = 7), soft and stiff Mesenchymal OV21 (soft: purple dashed line, n = 13; stiff: light red line, n = 9) and Non-Mesenchymal OV33 (soft: light blue dashed line, n = 15) tumors. Data are expressed as percentages of tumor area rather than in bins in order to compensate for the increasing number of pixels obtained as tumors grow. (**J**) Correlation plot between stiffness value of each pixel and distance from the tumor barycenter in Mesenchymal OV26 (soft n = 8; stiff: n = 8) and OV21 (soft n = 13; stiff n = 9) and Non-Mesenchymal OV33 (soft n = 15) tumors. Correlation coefficients *σ* and *P* value are based on Spearman’s rank correlation test.

Based on stromal signature characterizing Mesenchymal HGSOC, we hypothesized that stromal accumulation in these tumors could be associated with an elevated stiffness. To address this question in vivo, we benefited from three distinct PDX mouse models, two (OV26 and OV21) derived from two Mesenchymal HGSOC patients and one (OV33) from Non-Mesenchymal HGSOC patient. Interestingly, these PDX models fully recapitulated histological and molecular properties of their original human tumors (Fig. 1A,B), thereby confirming previous studies^34,41,71–73^. Indeed, the PDX models exhibited similar stromal and epithelial features as those observed in the corresponding initial human tumors (Fig. 1A). Moreover, the transcriptomic profiling of xenografted tumors clustered with the corresponding human HGSOC subtype (Fig. 1B), therefore confirming the reliability of OV26 and OV21 PDX models as Mesenchymal HGSOC, and the OV33 PDX model as Non-Mesenchymal one. We used shear wave elastography (SWE) as a non-invasive technique to quantify tumor stiffness over time. Briefly, supersonic SWE is based on the generation of shear waves, by an acoustic radiation force from an initial focused ultrasonic beam, into the targeted tissue. Stiffness of the tissue is then linked with shear waves speed and mapped by ultrafast ultrasound imaging. Using the ultrafast imaging device (Aixplorer), we could choose two different acquisition modes, penetration or resolution, for stiffness measurements^63^. Even if the two modes gave highly correlated tumor stiffness values (Supplementary Fig. 1A), we chose the resolution mode as it showed less noise than the penetration mode, the threshold value separating noise/signal being defined using a coupling gel, which is not supposed to generate any SWE signal (Supplementary Fig. 1B, see also “Methods” for technical details). Using this methodology, we first observed that tumor stiffness increased upon tumor growth in the two Mesenchymal HGSOC PDX models, while Non-Mesenchymal HGSOC PDX tumors remained mostly soft as they grew (Fig. 1C,D). Indeed, stiffness maps of representative tumors (Fig. 1C) and corresponding stiffness curves over time (Fig. 1D) showed that tumor stiffness significantly increased and reached 120 to 140 kPa over time in Mesenchymal HGSOC, while it remained low (not higher than 60 kPa) in Non-Mesenchymal tumors (Fig. 1D). In few cases, we observed a new tumor nodule emerging from a stiff Mesenchymal tumor (Supplementary Fig. 1C). Interestingly, the new nodule—of little size—was softer than the established initial tumor (Supplementary Fig. 1C), thereby confirming that tumor stiffness was associated with tumor growth in Mesenchymal HGSOC and that two regions, in close contact, could exhibit distinct stiffness. As we observed a stiffness signal in the skin surrounding the tumor (Fig. 1C), we wanted to make sure that this signal was not interfering with tumor stiffness measurements. We thus performed SWE measurement on soft and stiff tumors from Mesenchymal-OV26 PDX model, with or without the surrounding skin (Supplementary Fig. 1D–F) and confirmed the absence of interference between skin and tumor stiffness signals (Supplementary Fig. 1D,E). Moreover, to test whether the skin signal could be an artifact due to artificial acceleration of shear waves in the skin or a biologically relevant stiffness signal coming from tumor pressure, we injected an ultrasound gel between the skin and the tumor on one side in order to release tumor pressure (Supplementary Fig. 1F). By this way, we saw that the gel surrounding skin was clearly softer (5 kPa) than the tumor-surrounding skin (70 kPa) (Supplementary Fig. 1F), suggesting that the stiffness signal in the skin was not an artifact but the consequence of the pressure exerted by the tumor.

We next used this validated system for measuring stiffness in vivo (Fig. 1E–J). We first confirmed that tumor area measured by SWE technology was indicative of tumor volume assessed by a classic method (Supplementary Fig. 1G). Based on this method, we observed a strong correlation between mean tumor size, as assessed by tumor surface area imaged in the transverse plan (see “Methods”), and mean tumor stiffness in the two Mesenchymal HGSOC PDX models (Fig. 1E,F), while this correlation was lower in Non-Mesenchymal HGSOC (Fig. 1G). In addition, mean tumor stiffness progression over time was significantly higher in Mesenchymal HGSOC compared to Non-Mesenchymal tumors (Fig. 1H). Importantly, this was not associated with tumor growth rate, as Mesenchymal-OV26 tumors showed the most elevated stiffness but a growth rate as low as Non-Mesenchymal tumors (Supplementary Fig. 1H,I), suggesting that other properties than proliferation are important for tumor stiffness in Mesenchymal HGSOC. Finally, in line with stiffness variations in Mesenchymal HGSOC, we could distinguish soft (0 to 40 kPa) and stiff (0 to 120 kPa) tumors in Mesenchymal HGSOC, while all Non-Mesenchymal tumors remained soft (0 to 40 kPa) (Fig. 1I). Interestingly, in stiff Mesenchymal tumors, stiffness was higher at the center compared to the periphery, with more than 70 kPa decrease from the center towards the edge of the tumor (Fig. 1J). In contrast, in soft Mesenchymal tumors, stiffness remained low from the core to the periphery (Fig. 1J). Similarly, Non-Mesenchymal tumors were homogeneously soft at both center and periphery (Fig. 1J). Taken as a whole, these data show that human HGSOC exhibit distinct stiffness depending on their molecular subtype. Mesenchymal HGSOC show a gradual increase in stiffness upon growth particularly at their center, while Non-Mesenchymal HGSOC remain homogeneously soft, suggesting that stiffness in Mesenchymal HGSOC might be linked to tumor composition remodeling and specific molecular signaling.

### Myofibroblast content increases upon stiffening in Mesenchymal HGSOC

**Figure 2:**
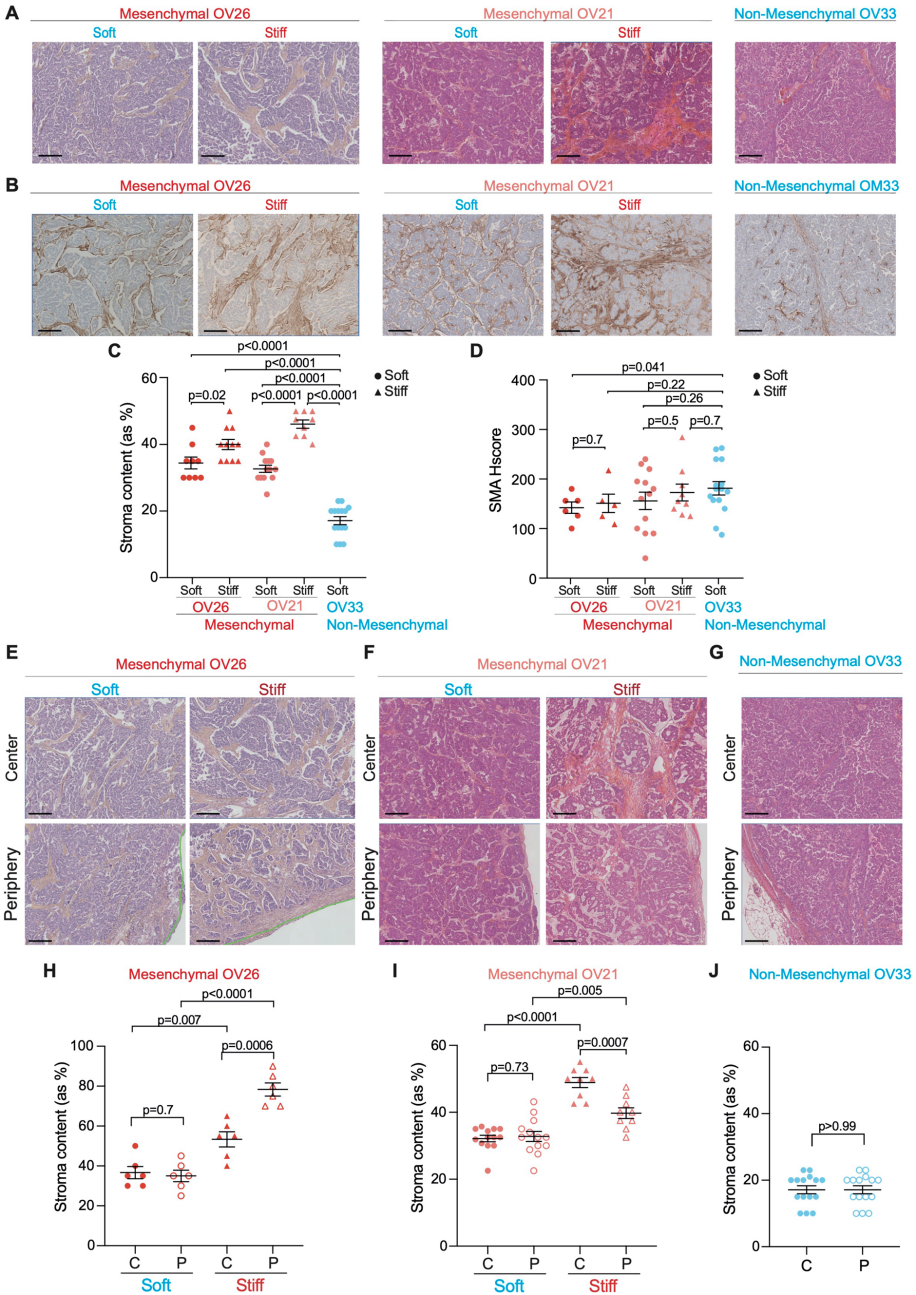
Tumor stiffness is associated with high myofibroblast content in Mesenchymal HGSOC. (**A**) Representative views of HES staining showing stromal (orange) and epithelial (pink) compartments in soft and stiff Mesenchymal and Non-Mesenchymal tumors. Scale bar, 200 μm. (**B**) Same as in (**A**) showing smooth muscle α-actin (SMA) immunostaining. (**C**) Scatter plot showing percentages of stroma in soft (dot) versus stiff (triangle) Mesenchymal (OV26: soft n = 9, stiff n = 11; OV21: soft n = 13, stiff n = 9) and Non-Mesenchymal (n = 15) HGSOC. Data are shown as mean ± S.E.M. *P* values from Mann Whitney test. (**D**) Same as in (**C**) showing SMA histological scores (Hscores). *P* values from Welch's t-test. (**E**–**G**) Same as in (**A**) at the center (top) and periphery (bottom) of soft (left) and stiff (right) Mesenchymal (**E**,**F**) and Non-Mesenchymal (**G**) HGSOC. (**H**–**J**) Scatter plot showing percentages of stroma at the center (C, plain) and periphery (P, empty) in soft (dot) versus stiff (triangle) Mesenchymal (OV26: soft n = 6, stiff n = 6) (**H**); (OV21: soft n = 13, stiff n = 9) (**I**) and Non-Mesenchymal (n = 15) (**J**) HGSOC. Data are shown as mean ± S.E.M. *P* values from Welch's t-test.

Based on the stromal-related signature defining Mesenchymal HGSOC^27–29,44–46,48–50^, we next characterized the histological features associated with tumor stiffness. As stiffness increases with tumor growth in Mesenchymal PDX models, we first examined if tumor stiffening could be linked to cancer cell proliferation by performing Ki67 immunohistochemistry (IHC) analysis. Epithelial ovarian cancer cells showed high levels of Ki67 but comparable proliferation rates between soft and stiff Mesenchymal HGSOC (Supplementary Fig. 2A,B), suggesting that stiffness was not associated with cancer cell proliferation. In addition, we measured necrosis using Hematoxylin and Eosin Saffron (HES) staining and found that the proportion of necrosis was almost negligible (less than 6% of tumor section) and equivalent in soft and stiff Mesenchymal tumors (Supplementary Fig. 2C,D). In contrast, we first observed that the proportion of stroma was significantly higher in Mesenchymal than in Non-Mesenchymal tumors (Fig. 2A,C), thereby confirming previous observations on Mesenchymal HGSOC^27–29,44–50^. Moreover, the global stromal content was also higher in stiff compared to soft Mesenchymal tumors (Fig. 2A,C), suggesting that tumor stiffness is associated with stroma content. We next assessed the proportion of smooth muscle α-actin (SMA)-positive fibroblasts (next referred to as myofibroblasts) in soft and stiff Mesenchymal tumors compared to Non-Mesenchymal tumors by performing SMA IHC analysis (Fig. 2B,D). We observed that fibroblasts exhibited high SMA staining and elevated histological score (Hscore) in the different HGSOC models (Fig. 2B,D), indicating that most fibroblasts are SMA+ in the three PDX models studied. Still, as the global fibroblast content was much higher in Mesenchymal HGSOC compared to Non-Mesenchymal ones (Fig. 2C), these data confirmed that Mesenchymal PDX tumors showed higher proportion of myofibroblasts, as observed in human HGSOC^44^. As we observed distinct stiffness at center and periphery of stiff Mesenchymal tumors, we next tested whether this observation could also be related to distinct stroma content. Stroma accumulation in stiff compared to soft Mesenchymal tumors was detected in all parts of the tumor (Fig. 2E,F,H,I), and was higher than in Non-Mesenchymal tumors (Fig. 2G,J). In conclusion, these results show that tumor stiffness in Mesenchymal HGSOC is associated with an accumulation of myofibroblasts. This is consistent with the desmoplastic reaction occurring in Mesenchymal versus Non-Mesenchymal HGSOC.

### Collagen network remodeling is associated with tumor stiffness in Mesenchymal HGSOC

**Figure 3:**
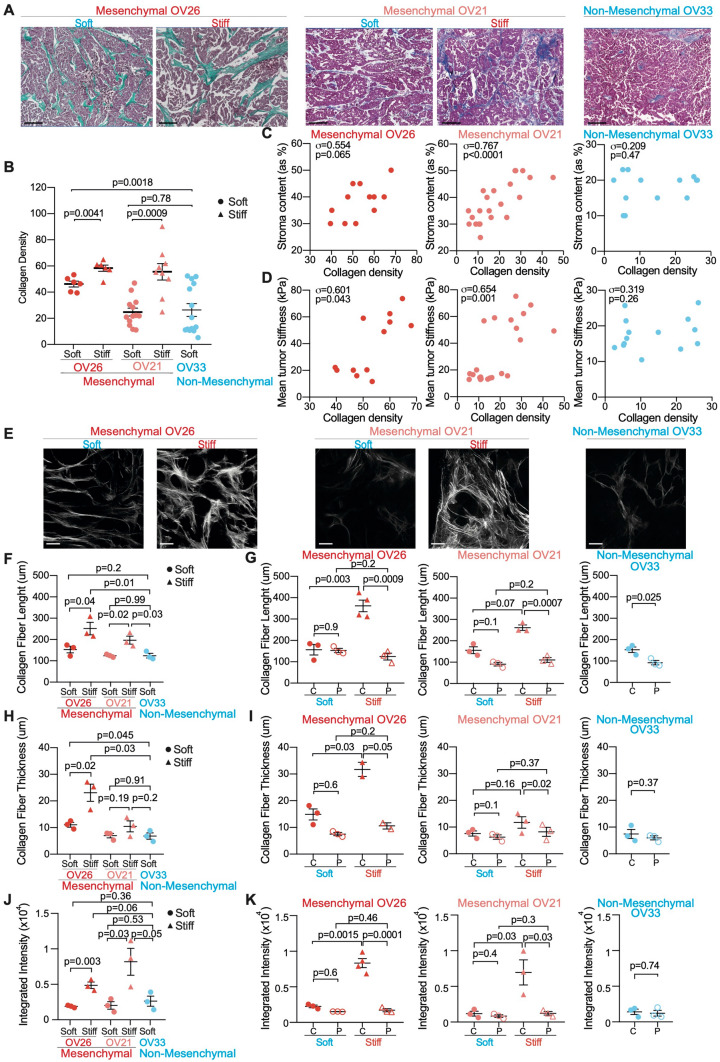
Tumor stiffening is correlated with collagen remodeling in Mesenchymal HGSOC. (**A**) Representative views of Masson’s trichrome staining showing nuclei (dark purple), cytoplasm (purple) and collagen (green/blue) in soft and stiff Mesenchymal and Non-Mesenchymal HGSOC. Scale bar, 200 μm. (**B**) Scatter plot showing collagen density in soft (dot) and stiff (triangle) Mesenchymal (OV26: soft n = 6, stiff n = 6; OV21: soft n = 13, stiff n = 9) and Non-Mesenchymal (n = 15) tumors. Data are shown as mean ± S.E.M. *P* values from Welch's t-test. (**C**) Correlation plots between stroma content (as percentage of total tumor section) and collagen density (evaluated using image J software^110^, see “Methods”) in Mesenchymal (OV26: n = 12; OV21: n = 22) and Non-Mesenchymal (n = 14) HGSOC. Correlation coefficients *σ* and *P* values are based on Spearman’s rank correlation test. (**D**) Same as in (**C**) between mean tumor stiffness (kPa) and collagen density. (**E**) Representative projected stack images of SHG signal in soft and stiff Mesenchymal and Non-Mesenchymal tumor sections. Scale bar, 100 μm. (**F**,**H**,**J**) Scatter plots showing collagen fiber length (**F**), thickness (**H**) and integrated density (see “Methods”) (**J**) in soft (dot, n = 3) and stiff (triangle, n = 3) Mesenchymal and Non-Mesenchymal (n = 3) tumor sections. Around 100 collagen fibers were measured in at least 10 representative regions per tumor. Data are shown as mean ± S.E.M. *P* values from Unpaired t-test. (**G**,**I**,**K**) Same as in (**F**,**H**,**J**) showing collagen fiber length (**G**), thickness (**I**) and integrated density (i.e. product of area and mean grey value) (**K**) at center (C, plain) or periphery (P) of soft (n = 3) and stiff (n ≥ 2) Mesenchymal and Non-Mesenchymal (n = 3) tumor sections. Around 100 collagen fibers were measured in at least 10 representative regions per tumor. Data are shown as mean ± S.E.M. *P* values are based on Paired t-test when comparing center versus periphery of the same tumor and Welch’s t-test when comparing soft versus stiff tumors.

Because collagen crosslinking has been shown to stiffen tumor matrix in breast cancer^60^, we wondered if collagen organization and structure could be remodeled in Mesenchymal HGSOC. We observed an increased collagen density in stiff Mesenchymal tumors compared to soft Mesenchymal and Non-Mesenchymal tumors, as assessed by Masson’s trichrome coloration (Fig. 3A,B). Interestingly, stroma content was correlated with collagen density in Mesenchymal HGSOC but not in Non-Mesenchymal tumors (Fig. 3C). In addition, collagen density was itself significantly correlated with stiffness specifically in Mesenchymal HGSOC (Fig. 3D). These data are consistent with Non-Mesenchymal tumors remaining soft as they grow (as shown Fig. 1C,D) and indicate that collagen density is associated with tumor stiffness in Mesenchymal HGSOC. We then used Second Harmonic Generation (SHG) microscopy on thick sections (400 μm) from the three HGSOC PDX models to analyze the structure of collagen fibers (Fig. 3E–K). Interestingly, not only collagen density (as shown Fig. 3A,B) but also collagen fiber' properties were distinct in the different HGSOC molecular subtypes analyzed (Fig. 3E–K). Moreover, we confirmed that collagen fibers were longer (Fig. 3F), thicker (Fig. 3H) and denser (Fig. 3J) in stiff than in soft Mesenchymal HGSOC, or compared to Non-Mesenchymal ones, as assessed by SHG integrated density that indicates the robustness of collagen local reorganization. Importantly, collagen quality differences between soft and stiff Mesenchymal tumors were driven by collagen fiber features at the center of stiff tumors (Fig. 3G,I,K). Indeed, collagen fibers were significantly longer (Fig. 3G), thicker (Fig. 3I) and denser (Fig. 3K) at the center of stiff Mesenchymal tumors compared to their periphery. In contrast, this was not the case in soft tumors (Fig. 3G,I,K). Thus, no difference in collagen quality was detected neither between tumor core and periphery of soft Mesenchymal, nor in Non-Mesenchymal tumors. This is consistent with the stiffness gradient from center to periphery that we only observed in stiff Mesenchymal tumors (as shown Fig. 1J). Thus, in addition to myofibroblast content, these results indicate that collagen network features are associated with tumor stiffness and HGSOC molecular subtype. Indeed, Non-Mesenchymal HGSOC, as well as soft Mesenchymal tumors, exhibit a diffuse, loose and relaxed collagen network, while stiff Mesenchymal tumors are characterized by an increase in long, thick and dense collagen fibers mainly at their core. The differential collagen remodeling between the center and the periphery of stiff Mesenchymal tumors might explain—at least in part—their stiffness heterogeneity.

### MEK is activated in mesenchymal HGSOC upon tumor stiffening

**Figure 4:**
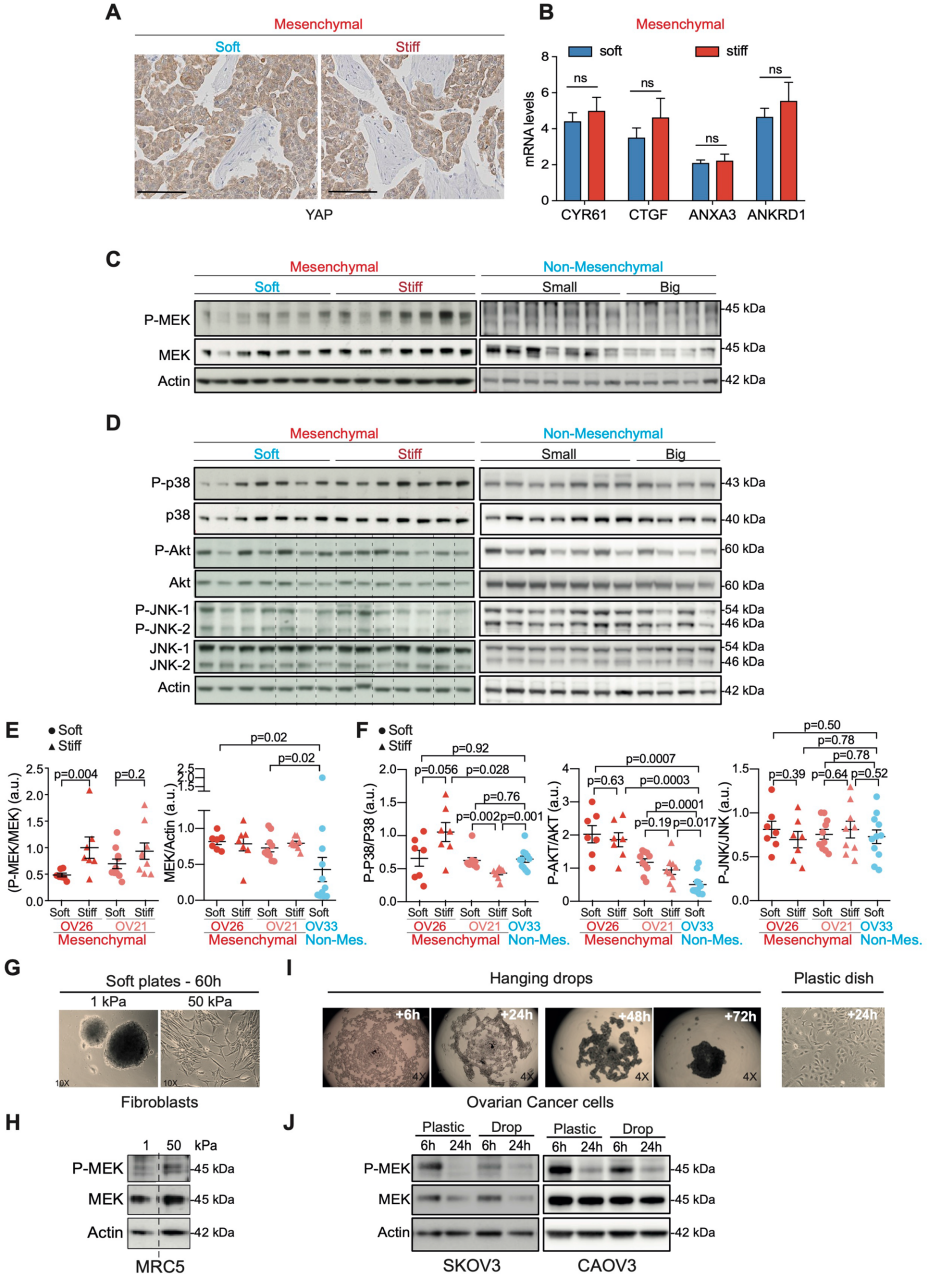
MEK is activated upon tumor stiffening of mesenchymal HGSOC. (**A**) Representative views of YAP staining in soft (left) and stiff (right) Mesenchymal OV26 tumors. Scale bar: 100 μm. (**B**) Bar plots showing CYR61, CTGF, ANXA3 and ANKRD1 mRNA expression levels normalized to cyclophilin A in soft (n = 6) and stiff (n = 6) tumors. Data are shown as mean ± S.E.M. *P* values from Student’s t-test. (**C**) Representative western blots showing the phosphorylated form (P-) and the total protein levels of MEK in soft (n = 7) *versus* stiff (n = 7) Mesenchymal (OV26) and Non-Mesenchymal (OV33) (n = 12) HGSOC. (**D**) Same as in (**C**) for P38, AKT, JNK-1 and JNK-2 in soft (n = 7) *versus* stiff (n = 7) Mesenchymal OV26 and Non-Mesenchymal (OV33) (n = 11) tumors. Dashed lines are used to delineate different parts of two different gels run, blotted and revealed at the same time, with the same time of exposure. (**E**) Scatter plots of P-MEK/MEK and MEK/Actin ratios from soft (dot) and stiff (triangle) Mesenchymal (OV26: soft n = 7, stiff n = 7; OV21: soft n = 10, stiff n = 9) and Non-Mesenchymal (OV33, n = 12) tumors, as assessed by densitometry analysis of western blots shown in (**C** and Supplementary Fig. 2E). Data are shown as mean ± S.E.M. *P* values from Welch's t-test (left panel) and Mann–Whitney test (right panel). (**F**) Same as in (**E**) for P-P38/P38, P-AKT/AKT and P-JNK/JNK ratios (but n = 11 for OV33 Non-Mesenchymal tumors). (**G**) Representative images of human MRC5 fibroblasts, cultured 60 h either on soft (1 kPa—left) or stiff (50 kPa—right) polyacrylamide hydrogels. (**H**) Representative western blots showing P-MEK and MEK protein levels from MRC5 cells cultured as described in (**G**). Dashed line is used to delineate different parts from the same gel. (**I**) Representative images of ovarian cancer cells cultured in hanging drops for 6 h to 72 h or on plastic dish for 24 h. (**J**) Representative western blots showing P-MEK and MEK protein levels from SKOV3 and CAOV-3 ovarian cancer cell lines cultured 6 h or 24 h either on plastic plate (stiff) or in hanging drops (soft). Actin is used as an internal control for all protein loadings.

In order to decipher the mechanism by which stiffness may modulate Mesenchymal HGSOC growth, we tested the activation state of several known pro-tumorigenic signaling pathways in soft and stiff Mesenchymal tumors. YAP and TAZ co-transcription factors being major mechano-transducers^51,64,74–78^ and markers of bad prognosis in human ovarian cancers^79^, we first analyzed YAP localization by IHC as well as the expression of its target genes in soft and stiff Mesenchymal tumors (Fig. 4A,B). However, YAP protein remained mainly cytoplasmic in both soft and stiff tumors (Fig. 4A) and we did not observe any differential expression of YAP target genes (including CYR61, CTGF, ANXA3, ANKRD1) in these different tumors (Fig. 4B), suggesting that YAP does not exert a key role in stiffness in Mesenchymal HGSOC. We previously demonstrated that MEK kinase is constitutively activated in 50% of human HGSOC through stabilization of its upstream regulator MAP3K8/COT^34^. Interestingly, here we found that MEK was significantly activated in stiff compared to soft Mesenchymal tumors (Fig. 4C,E and Supplementary Fig. 2E). Moreover, we observed that the total MEK protein level became almost undetectable upon growth in Non-Mesenchymal HGSOC, making MEK phosphorylation and activation impossible in these tumors (Fig. 4C,E). In contrast to MEK, AKT, P38 and JNK pathways were not consistently associated with stiff conditions in HGSOC (Fig. 4D,F). Thus, stiffness is mainly associated with MAPK/MEK pathway in HGSOC rather than with any other major pathways deregulated in cancer.

To investigate if MEK activation resulted from stiffness variation and not from tumor growth, we mimicked stiffness conditions in vitro using two different techniques: first, polyacryamide hydrogels (soft plates) that recapitulate stiffness variations observed in HGSOC; and second, hanging drops that exhibit low stiffness compared to plastic dishes (Fig. 4G–J). As MEK activation observed in stiff Mesenchymal tumors could result from cancer cells and/or stromal cells, we analyzed the impact of stiffness on MEK activation in vitro both in ovarian cancer cells and fibroblasts. As expected, fibroblast cell line (MRC5) cultured on 1 kPa hydrogel and two different ovarian cancer cell lines (SKOV3 or CAOV-3) cultured in hanging drops were not able to adhere to the matrix and progressively formed multicellular spheroids (Fig. 4G,I Left). In contrast, fibroblasts cultured on 50 kPa hydrogels and ovarian cancer cells plated on plastic dishes were able to adhere to the substrate and adopted a flat and elongated morphology (Fig. 4G,I Right). Consistent with what we observed in stiff Mesenchymal HGSOC (Fig. 4C,E), MEK phosphorylation was also increased in culture conditions mimicking stiffness, both in fibroblasts and ovarian cancer cells (Fig. 4H,J). Thus, these data indicate that MEK is activated in epithelial and stromal cells concomitantly to tumor stiffness increase both in vivo and in vitro.

### A stromal metabolic switch occurs upon stiffening in Mesenchymal HGSOC

**Figure 5:**
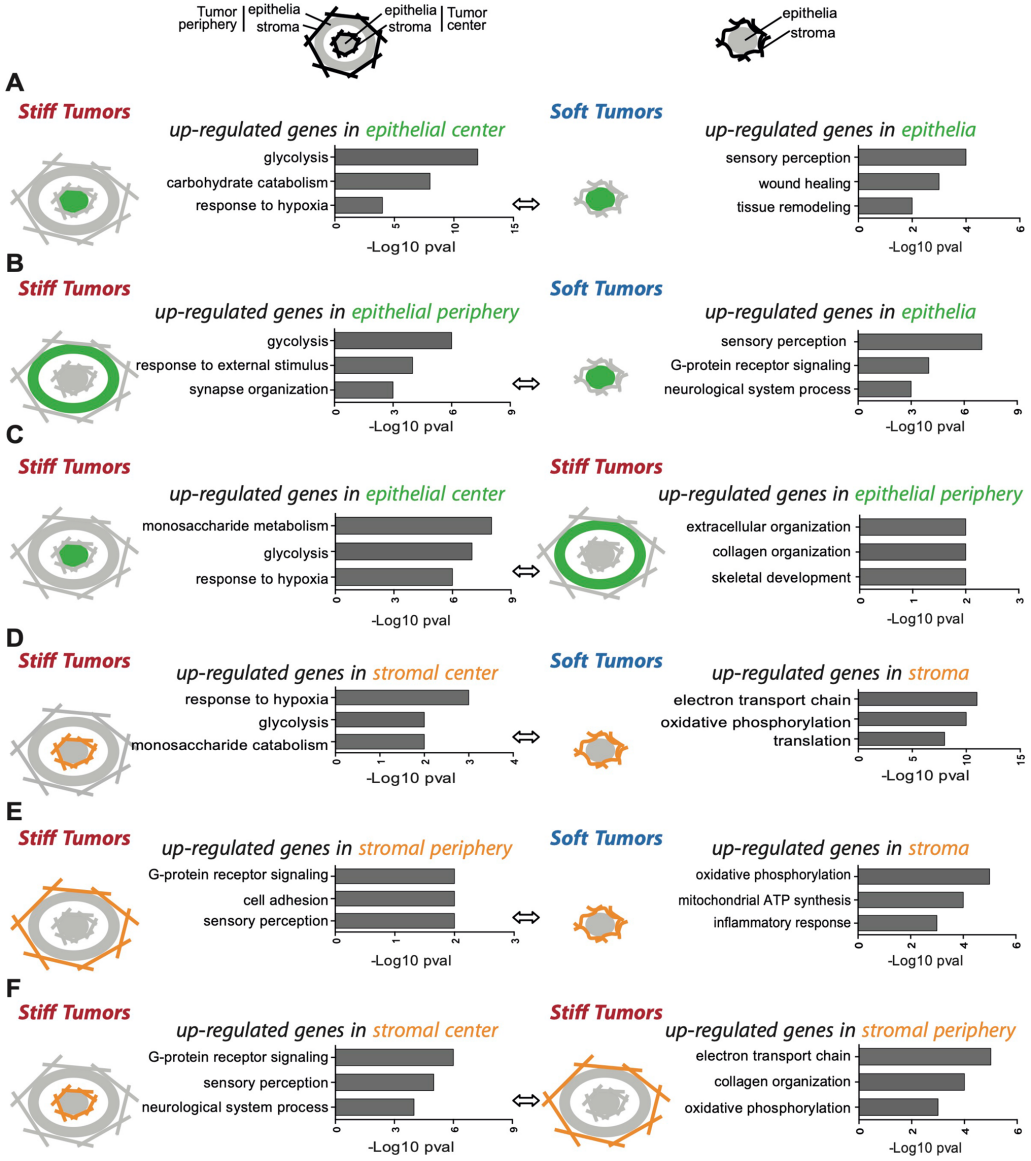
A metabolic switch occurs along tumor stiffening, in Mesenchymal HGSOC. (**A**–**F**) Representative pathways up-regulated in different tumor stiffness conditions and tumor localizations, as indicated by the different schemes. A plain circle (epithelial compartment) surrounded by wavy lines (stromal compartment) represents soft tumors. A plain circle (epithelial compartment) surrounded by straight lines (stromal compartment) illustrating matrix contraction, represents the core of stiff tumors. A thick circle line (epithelium) surrounded by straight lines (stroma) represents the periphery of stiff tumors. Orange represents the stromal compartment localization and green the epithelial compartment localization when performing differential analysis in stiff *vs.* soft tumors or within stiff tumors. Stromal and epithelial genes expression are based on murine MTA 1.0 or human HTA 2.0 microarrays, respectively. T-test was used to compare gene expression between two conditions. For each condition, the top 3 DAVID biological pathways defined by the up-regulated gene lists are represented. We analyzed 3 soft tumors, 5 stiff tumors both at their periphery or at their center. *P* values are presented as – Log 10. (**A**) Genes up-regulated in the epithelium at the center of stiff tumors (left) compared to genes up-regulated in the epithelium of soft tumors (right). (**B**) Genes up-regulated in the epithelium at the periphery of stiff tumors (left) compared to genes up-regulated in the epithelium of soft tumors (right). (**C**) Genes up-regulated in the epithelium at the center (left) compared to the periphery (right) of stiff tumors. (**D**) Genes up-regulated in the stroma at the center of stiff tumors (left) compared to genes up-regulated in the stroma of soft tumors (right). (**E**) Genes up-regulated in the stroma at the periphery of stiff tumors (left) compared to genes up-regulated in the stroma of soft tumors (right). (**F**) Genes up-regulated in the stroma at the center (left) compared to the periphery (right) of stiff tumors.

To get insights into epithelial and stromal molecular signatures associated with tumor stiffness in Mesenchymal HGSOC, we performed transcriptomic analysis in soft and stiff tumors (Fig. 5), considering both tumor center and periphery, as stiff Mesenchymal HGSOC exhibit distinct stiffness from the core to the edge (as shown Fig. 1J). To do so, we took advantage of PDX using species-specific micro-arrays, as the murine stroma replaces the human one in these models. We first compared transcriptomic signatures detected in the epithelial compartment of soft and stiff Mesenchymal tumors (Fig. 5A–C). In agreement with the well-known Warburg effect occurring in cancer cells upon tumor growth^80–82^, the epithelium of stiff HGSOC, whether it was located at the center or at the periphery, exhibited glycolytic and hypoxic signatures compared to soft tumors (Fig. 5A,B, Left). These glycolytic and hypoxic signatures of stiff tumors were not related to cellular proliferation, as soft and stiff tumors showed similar proliferation rate (Supplementary Fig. 2A,B). When we restricted our analysis to stiff tumors, we found that the epithelium at the center was also significantly enriched in glycolytic metabolism compared to periphery (Fig. 5C, Left), consistent with a higher stiffness at the center. As opposed to metabolic regulation in stiff tumors, we found that the epithelium of soft tumors exhibited an enhanced expression of genes involved in sensory perception (G-protein coupled receptors), tissue remodeling and wound healing (Fig. 5A,B, Right). Similarly, the periphery of stiff tumors showed signatures of ECM and collagen organization (Fig. 5C, Right), thereby confirming, at transcriptional level, the increased collagen density and ECM remodeling we observed (Fig. 3). We thus next compared the stroma of soft and stiff Mesenchymal tumors (Fig. 5D–F). While the stroma at the center of stiff tumors was glycolytic (as we observed for the epithelium) (Fig. 5D, Left), we found an upregulation of genes involved in oxidative phosphorylation (OXPHOS) metabolism in the stroma of soft tumors (Fig. 5D,E, Right). Interestingly, the OXPHOS signature of the stroma was found both in soft tumors and at the periphery of stiff tumors (Fig. 5F, Right), which was consistent with stiff tumors being soft at their periphery. When looking at global Electron Transport Chain (ETC) protein level by western blot in soft and stiff Mesenchymal tumors without distinguishing the epithelium or the stroma, we did not observe any difference, possibly because of opposite metabolic signature between the glycolytic epithelium and the oxidative stroma upon tumor stiffening. Taken as a whole, these results reveal that tumor stiffening is associated with a glycolytic switch not only in epithelial cancer cells (as expected based on Warburg’s effect) but also in stromal cells. Interestingly, this effect is restricted to the central part of stiff tumors and is not detected at the periphery. Indeed, the periphery remains softer than the core of stiff tumors, which accumulates high density of thick and long collagen fibers. Moreover, at periphery, while epithelial cells are glycolytic, stromal cells show an OXPHOS metabolism, thereby suggesting a different metabolism between stromal and epithelial compartments during tumor stiffening.

## Discussion

HGSOC with Mesenchymal signature is associated with poor patient survival and chemotherapeutic resistance^27–30,32–34,43^. This signature is mainly composed of stromal genes involved in ECM remodeling^29,31^. Interestingly, Mesenchymal HGSOC show high stromal content composed of myofibroblasts and ECM proteins, such as collagen and fibronectin^27–29,44–46,48–50^. Increased ECM deposition in tumor microenvironment is one of the major causes of tumor stiffness. In ovarian tumors, not much is known about the impact of stiffness on tumor progression. By studying relevant PDX mouse models of both Mesenchymal and Non-Mesenchymal HGSOC, here we show that tumor stiffness is associated with HGSOC molecular subtype. Indeed, tumor stiffness highly increases with tumor growth in Mesenchymal HGSOC, while Non-Mesenchymal tumors mainly remain soft. Tumor stiffness is thus a central parameter in Mesenchymal HGSOC that is associated with major compounds involved in tumor progression. Indeed, we observed that tumor stiffness strongly correlates with tumor growth and is associated with increased myofibroblast content, high collagen deposition and remodeling, and MAPK/MEK pathway activation. Interestingly, we also observed a stiffness gradient from the center to the periphery of stiff Mesenchymal tumors that is associated with distinct collagen network and metabolic signatures in epithelial and stromal compartments. As a whole, our work reveals that Mesenchymal and Non-Mesenchymal HGSOC are distinct regarding tumor stiffness. The increased stiffness, which occurs only in Mesenchymal HGSOC upon tumor growth, might contribute to the aggressiveness of this specific HGSOC molecular subtype by inducing MAPK/MEK signaling pathway, stromal metabolic switch and matrix remodeling.

We chose PDX mouse models to assess the impact of stiffness on HGSOC stromal and molecular features in vivo. In these models, HGSOC tumor fragments coming from patient’ biopsies were grafted into the interscapular fat pad of immunodeficient mice. This specific location constitutes a rich nutritional and vascularized environment that is particularly beneficial for successful grafting and tumor growth. Although more difficult to establish than mouse models with injected cell lines, PDX models fully recapitulate histological and molecular features of the initial human tumor, as previously published by us and others^34,41,71–73^, thus representing one of the major advantages of these mouse models. As opposed to orthotopic xenograft and syngeneic mouse models, PDX models have distinct tumor location than the original tumor and tumor microenvironment lacks T lymphocytes. Nevertheless, using PDX models to investigate how myofibroblast content, ECM remodeling and collagen fibers structure contribute to tumor stiffness in Mesenchymal versus Non-Mesenchymal tumors constitute a relevant model.

We chose an innovative technology called Supersonic shear wave elastography, to follow stiffness in vivo in a non-invasive way. Although this ultrasound-imaging tool is already used in clinical practice to detect malignant tumors^57,83–86^, its application to investigate stiffness and its pathological consequences in preclinical animal models is quite recent^63,87–89^. Our study provides new evidences of its validity for ovarian cancer. Indeed, by comparing various properties of relevant mouse models from both Mesenchymal and Non-Mesenchymal HGSOC, we found that tumor stiffness strongly correlates with tumor growth in PDX models of Mesenchymal HGSOC. This result is consistent with previous studies in other mouse models, showing a high correlation between tumor stiffness and maximum tumor diameter^63,90,91^. Indeed, stiffness increases upon tumor growth in PDX model of luminal B breast cancer and is strikingly associated with a fibrotic reaction and a desmoplastic stroma^63^. In line with these observations, we found that tumor stiffness is associated with a high content in myofibroblasts, consistent with previous study in human HGSOC^27–29,44–50^. Interestingly, it has been shown that stroma have a strong influence on tumor mechanical propertie^55,59,78^. Myofibroblasts modulate tumor stiffness by secreting factors that bind to and remodel the ECM, such as matrix metalloproteinases and serpin proteins^52,92,93^. Importantly, we observed a correlation between high myofibroblast content and elevated collagen density, which correlates with tumor stiffness. This indicates that myofibroblast accumulation might induce ECM remodeling that in turn influences tumor stiffness in Mesenchymal HGSOC. Indeed, tumor stiffness directly depends on ECM remodeling, as well as on the mechanical dialogue between intracellular mechano-sensors and cell-generated forces^54,61^. Notably, increased collagen deposition, collagen cross-linking and collagen fibers reorientation were shown to increase tumor stiffness^60,94,95^. Using second harmonic generation microscopy, we demonstrated that tumor stiffness is associated with a reorganization of the collagen network into long and thick collagen fibers. These data are in agreement with previous works showing that collagen remodeling accompanies and enhances tumor stiffening^60,94–97^. Interestingly, we observed that big Mesenchymal HGSOC display stiffness heterogeneity, between the core (stiff) and the periphery (soft), which is accompanied by a distinct collagen network density and quality. Indeed, collagen fibers are denser, longer and thicker at the core than at the periphery of stiff Mesenchymal HGSOC. Importantly, collagen quantity and reorganization into thick and linearly oriented fibers correlate with tumor progression and clinical outcome in breast cancer^98^. When oriented, collagen fibers help the directional migration of tumor cells towards blood vessels, thereby facilitating metastasis^55,99,100^. In our model, the highly reticulated collagen network observed in stiff tumors might contribute to the aggressiveness and poor outcome of Mesenchymal HGSOC patients. The reason why the periphery of big tumors remains soft is still unclear. Although this could be a transient phenomenon, we can hypothesize that tumor periphery represents the exit door for cancer cells to leave the primary mass and colonize other organs, and its low stiffness may help them to do so. In that sense, an increase in the malignancy of metastatic ovarian cancer cells, including migration and invasion capabilities, was observed on soft matrices^67^.

Tumor stiffness promote tumor progression mainly by stimulating mechano-transduction pathways in epithelial cancer cells and in stromal cells^101^. Typically, high stiffness increases cancer cell migration and invasion through a RHO-ROCK-dependent actin remodeling and reciprocal actomyosin-mediated cell contractility^54,102^. In addition, rigid matrix can convert fibroblasts into contractile myofibroblasts, which produce more matrix components (including collagen, fibronectin, glycosaminoglycans) thereby remodeling the matrix. Through the activation of the mechano-transducer YAP, myofibroblasts are also able to increase matrix stiffening, creating a positive feedback-loop, and sustaining tumor progression^52^. We identified MEK as a kinase activated during tumor stiffening. MEK activation in stiff HGSOC may not be specific of one tumor compartment but may arise in epithelial and stromal cells. MEK activation has already been involved in tumor aggressiveness, promoting cancer cell proliferation, migration and invasion^34,103^, suggesting that MEK could mediate the impact of stiffness in Mesenchymal HGSOC. ECM remodeling is involved in inducing almost all intracellular signaling pathways, including MAPK/MEK. Indeed, receptors such as integrins that are involved in cell matrix interaction are major mechano-sensors of the ECM. Once activated by specific ECM molecules they activate intracellular signaling complexes leading to the activation of MAPK and PI3K/Akt pathways in order to promote cell proliferation, differentiation and migration^104^. It is thus possible that ECM remodeling occurring upon stiffening in Mesenchymal HGSOC would lead to MEK activation and contribute to the aggressiveness of this subtype of ovarian cancer.

PDX models are notably characterized by the progressive replacement of the human stroma by the murine one^105–107^, but the stromal structure and composition is conserved^108,109^. This specific PDX feature gave us the opportunity to differentiate the stromal gene expression from the epithelial gene expression, by using either human or murine specific microarrays. Transcriptomic analysis between soft and stiff tumors allowed us to define stiffness-associated metabolic signatures, unveiling metabolic switches. Indeed, a glycolytic metabolism switch occurred in epithelial cells, which most probably reflects the Warburg’s effect. Stromal cells also displayed a metabolic switch from an oxidative phosphorylation metabolism to glycolysis. Interestingly, the stromal metabolic switch was not observed in the periphery of stiff Mesenchymal tumors, where the oxidative phosphorylation signature was still present, reinforcing the particularity of this region. Overall, our results suggest that stiffness could participate in the glycolytic switch in cancer and stromal cells. This is consistent with the metabolic reprogramming occurring both in cancer and stromal cells in response to stiffening in the tumor niche in a model of breast cancer^70^. The stiffness increase could be a way to have rapid and large-scale impact on cells, improving their survival in harsh conditions. The energy support they need, could be provided by the stromal cells through an oxidative phosphorylation metabolism.

## Conclusion

Our work gives new insights in favor of the existence of high tumor heterogeneity, based on mechanical regulations, conferring a high level of organization into tumor but also unveiling possible therapeutically targetable markers. We speculate that tumor stiffness could thus be an additional, and easy to introduce, predictive parameter for MEK inhibitors in Mesenchymal HGSOC, SWE being already available in clinic.

## Methods

### Cohorts of HGSOC patients

Institut Curie’s ovarian cancer microarray data set used for transcriptomic analyses is freely accessible in the Gene Expression Omnibus under the accession number GSE26193. Ovarian tumors were obtained from a cohort of 107 patients treated at the Institut Curie between 1989 and 2005. Ovarian tumor samples have been analyzed on Human Genome U133 Plus 2.0 microarrays (Affymetrix). Clinical features of the cohort have already been described in^29,34,41,44^. The project developed here is based on surgical tumor tissues, from the Institut Curie Hospital Group, that are available after histo-pathological analyses and not needed for diagnosis. There is no interference with the clinical practice. Analysis of tumor samples was performed according to the relevant national law providing protection to people taking part in the biomedical research. Their referring oncologist informed all patients included in our study that their biological samples could be used for research purposes and patients signed an informed consent of non-opposition. In case of oral or written patient's refusal, residual tumor samples were excluded from our study. Human experimental procedures for studies driven by Dr. F. Mechta-Grigoriou were approved by the Institutional Review Board and Ethics committee of the Institut Curie Hospital group (approval February 12, 2014), declared to the CNIL (Commission Nationale de l’informatique et des Libertés) (N° approval: 1674356 delivered March 30, 2013) and authorized by the Committee of Person Protection (RIPH 20.01.10.44622).

### Patient-derived xenograft (PDX) models of Mesenchymal and Non-Mesenchymal HGSOC

The OV26 and OV21 PDX models of Mesenchymal HGSOC and the OV33 PDX model of Non-Mesenchymal HGSOC were established at Institut Curie (Paris, France) with patient consent according to the relevant national law on the protection of people taking part in biomedical research. These PDX models have already been used in^34,41,44^. Briefly, human tumor fragments of 30–60 mm^3^ were grafted into the interscapular fat pad of 6-weeks-old Swiss nude female mice, under avertin anesthesia (n ≥ 11). Human tumor xenograft reached a volume of 40–120 mm^3^ approximately 20 days, 25 days and 30 days after grafting for OV21, OV26 and OV33 PDX models, respectively. Tumor size was evaluated by measuring two perpendicular diameters of tumors with a caliper twice a week. Individual tumor volumes were calculated as *(V)* = *a* × *b*^2^/2 with a being the major and b the minor diameter. This was followed by tumor imaging twice a week, using Aixplorer and evaluation of the tumor area with SonicSoftware tool (see below). All protocols involving mice and animal housing were in accordance with institutional guidelines as proposed by the French Ethics Committee (CEEA-IC 118). Establishment and maintenance of PDX models at Institut Curie have been approved by the French ministry of Higher Education, Research and Innovation (agreement number #2163.02). Moreover, projects using mouse models of breast and ovarian tumors from the “Stress and Cancer” lab headed by Dr. F. Mechta-Grigoriou have received authorization from the French ministry of Higher Education, Research and Innovation (agreement number #02300.02). The study was carried out in compliance with the ARRIVE guidelines.

### Supersonic shear wave elastography in vivo

Supersonic SWE is based on the generation of shear waves, by an acoustic radiation force from an initial focused ultrasonic beam, into the targeted tissue. Stiffness of the tissue is then linked with shear waves speed and mapped by ultrafast ultrasound imaging. Supersonic SWE measures quantitatively tissue stiffness in vivo. Due to high water content of soft biological tissues, including ovarian tumors, one can assume that the medium exhibit a local constant density ρ and an incompressible and isotropic structure^87,111^. Shear waves speed vs is thus linked to the Young’s modulus E (in kPa) expressing the targeted tissue stiffness via the formula: E = 3ρ × vs^2^. We have chosen the option of subcutaneous human tumor engraftment and SWE for several reasons. First, we could easily and reliably follow tumor volume over time. Second, as mentioned above, subcutaneous-engrafted human HGSOC in PDX mouse models mirror initial patient tumor features (Fig. 1A,B). Third, stiffness measurements are highly improved when performed on tissues located just underneath the skin, as SWE sensitivity is quite limited in deep body tissues due to acoustic radiation force attenuation by tissue penetration. This is particularly the case for tumors maintained at relatively small sizes for ethical reasons in line with animal care. Tumor stiffness measurement using SWE on PDX model has already been described in^63,88^. Briefly, ultrasound tumor measurements were performed twice a week during tumor growth, starting with a minimum tumor volume of 30–60 mm^3^. Images were acquired using a 15 MHz high frequency ultrasound probe (SL22-7) from an ultrafast imaging device (Aixplorer, Supersonic Imagine, Aix-en-provence, France). Upon isoflurane anesthesia (3% for induction and 1.5% for maintenance) mice were placed on a heating pad at 37 °C (THM150, Indus Instruments) in supine/prone position and the tumor area was carefully covered with ultrasound gel to minimize air bubbles and to reduce artifact signal. The probe was directly applied on the gel with a motorized controlled arm. All acquisitions were performed in the transverse plan to exclude spinal column-mediated interferences. The tumor shape being spherical, we acquired B-mode images in the transverse plan and select the position showing the largest tumor diameter. We then acquired SWE images using the preset resolution mode. This setting, that maximizes resolution of stiffness maps, provided the most robust and specific stiffness maps as opposed to the SWE penetration mode. Indeed, even if the two modes gave highly correlated tumor stiffness values, as mentioned above, tumor stiffness maps obtained from the resolution mode showed less noise than the penetration mode, the threshold value separating noise/signal being defined using a coupling gel, which is not supposed to generate any SWE signal. We set the stiffness scale from 0 kPa (in purple) to 120 kPa (in red) as it enabled us to visualize well stiffness evolution during tumor growth. Upon activation of SWE mode, we waited few seconds for stiffness map stabilization and then recorded a 5 s cine loop (SWE maps’ frame rate being one image per second). We repeated the acquisition procedure, changing the ultrasound gel and repositioning the probe at the largest tumor diameter 3 times for each tumor, at each time point, in order to minimize variations due to probe positioning.

### Stiffness maps measurement

The built in SonicSoftware tool (Supersonic Imagine, Aix en Provence, France) was used to perform stiffness measurements: from each cine loop recorded, we chose one representative B-mode/SWE image, based on limited signal from the gel, as it corresponds to noise. We drew the tumor contour using B-mode image. The software provided the minimum, maximum, mean and standard deviation of stiffness value inside this Region Of Interest (ROI). We thus determined the global stiffness of each tumor by averaging the mean stiffness values obtained with the software of over 3 SWE acquisitions performed for each tumor at each time point. Soft and stiff Mesenchymal tumors were imaged and removed either at a global mean stiffness ranging from 8.5–22.6 kPa (soft OV26) and 11.8–20 kPa (soft OV21) or 54.6–78 kPa (stiff OV26) and 42.5–75.2 kPa (soft OV21), respectively. Non-Mesenchymal tumors (OVM033-BC) were imaged and removed at a global mean stiffness ranging from 11.5 to 31.8 kPa. In order to evaluate the impact of the skin on tumor stiffness, we either removed the skin above the tumor on anaesthetized mice using isoflurane and intraperitoneal injection of meloxicam (1 mg/kg) (Metacam) with ultrasound gel directly applied on the tumor; or we injected ultrasound gel at the interface between tumor and skin. In both cases, mice were sacrificed immediately after imaging procedure.

### Stiffness maps analysis

To further analyze the stiffness distribution within the tumor area, we took advantage of the Quantitative DICOM software option (provided by the manufacturer) to retrieve the stiffness value in each pixel from each selected image. Indeed, this option allowed for each single acquisition frame exported in DICOM format the addition of the original stiffness map in a proprietary DICOM field. A Matlab dedicated software was programmed to retrieve from DICOM files both tumor contour (from B-mode image) and stiffness values of each pixel inside the corresponding area. Histograms of stiffness from pixels inside the ROI can be calculated then. This map also allowed calculating the distance of each pixel inside the ROI from the tumor barycenter and relating it to its stiffness value.

### Immunohistochemistry staining on tumors from mesenchymal and non-mesenchymal HGSOC PDX models

Serial sections of paraffin-embedded tumors (3 μm) were stained either with HES (Hematoxylin–Eosin-Saffron), Masson’s trichrome, Ki67, α-SMA or YAP antibody. Masson’s trichrome staining was performed either using the trichrome stain (Masson) kit (Sigma-Aldrich, #HT15-1KT) according to Manufacturer's instructions on paraffin embedded tumor sections from OV21 and OV33 PDX models, or tumor sections from OV26 PDX model were incubated with hematoxylin for 10 min after deparaffinization and rehydration, then with acid fuchsin-ponceau solution (1v/2v mix of 1% acid fuchsin and 1% xylidine-ponceau) for 5 min, then with 1% molybdatophosphoric acid solution, then with 1% light green for 5 min and finally with 1% acetic acid for 5 min. Collagen density was analyzed using Image J software^110^. Pictures from Masson's trichrome stained tumor sections were taken at low magnification (1×), using the Image Management Software from Philips Digital Pathology in order to get the whole tumor section in one image. After color deconvolution and identical threshold set up for all pictures analyzed (one per tumor), we used the freehand tool to select the area of the tumor that we calibrated in square millimeter, and we measured Integrated Density (product of area and mean grey value i.e. average of grey values for all pixels within the area of selection) considered as collagen density. For Ki67, α-SMA and YAP IHC, we used a streptavidin-peroxidase protocol (Vectastain ABC kit; Vecto Labs, #PK-6101) and the Autostainer 480 Labvision (Thermoscientific), as previously described^34,41,44^. In brief, paraffin embedded sections were incubated with specific antibody recognizing Ki67 (1:150; Dako, #M7240), α-SMA (1:400; Dako, #M0851) or YAP (1:400; SantaCruz, # sc-15407) in PBS solution at pH 7.6 containing 0.05% Tween 20 for 1 h, following unmasking in citrate buffer, pH 6 (Dako, #S2369), for 20 min at 97 °C. For quantification, 2 independent researchers blindly evaluated at least five distinct areas of each tumor. Histological score (Hscore) of Ki67 and YAP staining in epithelial cells or α-SMA staining in fibroblasts were given as a function of the percentage of positive cells multiplied by staining intensity (ranging from 0 to 4) in soft (n > 6) and stiff (n > 5) tumors.

### Second harmonic generation (SHG) microscopy and image analysis

For non-linear imaging, tumors were removed and immediately fixed in AFA (75% Ethanol, 2% Formol and 5% Acetic Acid) fixative for 12 h, and conserved in ethanol (70%) at 4 °C. Before imaging, tumors were washed twice with PBS, included in agarose gel, and cut in the transverse plan into 400 μm slices using a vibratome (Leica Microsystems Gmbh, Wetzlar, Germany). Upright stand Leica SP5 multiphoton microscope was used for tissue imaging (Leica Microsystems). A Ti:Sa Chameleon Ultra II (Coherent, Saclay, France) tuned at a wavelength of 810 nm was used as the laser source for second harmonic generation (SHG). The laser beam was circularly polarized using a quarter-wave plate in order to excite the slices isotropically regardless of the orientation of fibrillar collagen. Leica HC FLUOTAR VISIR 25×/0.95 W objective was used to excite and collect SHG. Signal was detected in epi-collection through a 405/15-nm bandpass filter, by NDD PMT detector (Leica Microsystems) with a constant voltage supply, at constant laser excitation power, allowing direct comparison of SHG intensity values. LAS software (Leica, Germany) was used for laser scanning control and image acquisition. Z-stack of 400 µm thickness and x–y mosaic reconstructions were captured and reconstructed by overlap stitching in a representative tumor. The collagen fibers structure was analyzed using homemade routines Image J software^110^ on maximum intensity projected stacks. To assess collagen fiber length, freehand lines were drawn along at least 100 fibrils per condition. Fiber thicknesses were obtained by applying a threshold on projected z-stack ROI and drawing straight lines across at least 100 fibrils per condition. Integrated density, which is the product of area and mean grey value of each pixel, indicating the robustness of collagen local reorganization, was obtained by summing up the pixel values in at least 10 representative ROI. As mentioned, the length, the thickness and the integrated density of each condition were determined by averaging all the results from each image’s ROI and from two to four different tumors.

### Epithelial and fibroblast cell lines and cell culture conditions

For our study, we chose representative fibroblast (MRC5, ATCC# CCL-171) and epithelial ovarian cancer (SKOV3, ATCC# HTB-77 and CAOV-3, ATCC# HTB-75) cell lines, in order to analyze the stiffness-mediated molecular effects in stromal and epithelial cells. Each cell line identity was tested by Short Tandem Repeat (STR) DNA profiling (Promega, #B9510) and tested for absence of mycoplasma contamination. Cells were propagated in DMEM (Gibco, ThermoFisherScientific # 11995) supplemented with 10% fetal bovine serum (FBS, Biosera #FB-1003/500), penicillin (100 U/ml) and streptomycin (100 μg/ml) (ThermoFisherScientific #15140122) in a humidified atmosphere of 5% (v/v) CO_2_ in air at 37 °C. MRC5 were cultured on soft plates for 60 h either on 1 kPa (Matrigen, # Softwell 6—1 kPa/Easy Coat/6 well plate) or 50 kPa (Matrigen, # Softwell 6–50 kPa/Easy Coat/6 well plate) polyacrylamide hydrogels coated with collagen type I rat tail (10 μg/ml; Institut de Biotechnologie Jacques Boy, #207050357). SKOV3 and CAOV-3 were cultured for 6 or 24 h either on plastic dish or in hanging drops. The hanging drop culture consist of resuspending 2 × 10^5^ SKOV3 cells or 3 × 10^5^ CAOV-3 cells in 1 ml cell culture medium, per condition, and distributing the whole cell suspension as 20 μl drops on the inner cover of a petri dish lid with an automated pipette. The lid is then inverted onto cell culture medium-filled bottom chamber and incubated in a humidified atmosphere of 5% (v/v) CO_2_ in air at 37 °C for the indicated times.

### Protein extracts from PDX tumors

PDX tumors removed from mice were snap-frozen in liquid nitrogen and store at − 80 °C. Tumors were incubated with boiling lysis buffer (50 mM Tris pH 6.8, 2% SDS, 5% glycerol, 2 mM dithiothreitol, 2.5 mM EDTA, 2.5 mM EGTA, 4 mM Na3VO4 and 20 mM NaF) supplemented with 2× Halt phosphatase inhibitor cocktail (Perbio #78420) and 1 tablet per 5 ml lysis buffer of Complete protease inhibitors, EDTA-free (Roche #05892791001) in 2 ml Eppendorf tubes containing two stainless beads (Qiagen, #69989). The tubes were inserted in a tissue lyser for 3 min at 30 Hz. Then, the lysates were incubated for 10 min at 95 °C, quickly spin down prior to 5 min sonication (Bioruptor, Diagenode Pico) and centrifuged for 10 min at 13,200 rpm at room temperature. The supernatant was transferred in a new Eppendorf tube, snap-frozen in liquid nitrogen and stored at − 80 °C.

### Protein extracts from cell lines

For SKOV3 and CAOV-3 cells cultured in hanging drops as well as for MRC5 cultured on 1 kPa hydrogels, since the cells form multicellular spheroids floating in the medium, we aspirated the medium with the spheroids. After centrifugation, the spheroids were resuspended directly into boiling lysis buffer (50 mM Tris pH 6.8, 2% SDS, 5% glycerol, 2 mM dithiothreitol, 2.5 mM EDTA, 2.5 mM EGTA, 4 mM Na3VO4 and 20 mM NaF) supplemented 2× Halt phosphatase inhibitor cocktail (Perbio #78420) and 1 tablet per 5 ml lysis buffer of Complete protease inhibitors, EDTA-free (Roche #05892791001). For mechanical dissociation, the spheroids were aspirated up and down through a 22 μm diameter needle. For SKOV3 and CAOV-3 cells cultured on plastic dishes as well as for MRC5 cultured on 50 kPa hydrogel, since cells adhere to the substrate, they were washed with cold PBS once and scraped with the above-mentionned boiling lysis buffer, transferred into an Eppendorf tube and incubated for 10 min at 95 °C. The protein extracts were then quickly spin down prior to 5 min sonication (Bioruptor, Diagenode Pico) and centrifuged for 10 min at 13,200 rpm at room temperature. The supernatant was transferred in a new Eppendorf tube, snap-frozen in liquid nitrogen and stored at − 80 °C.

### Western blot analysis of protein extracts from PDX tumors and cell lines

The “Stress and Cancer” lab headed by Dr. F. Mechta-Grigoriou has already published the following method^34^. Briefly, protein concentration was determined using BCA Protein Assay kit-Reducing Agent Compatible (Pierce Laboratories, ThermoFisherScientific, #23225). 10 μg of protein extract were loaded onto 4–12% polyacrylamide gels (Invitrogen, #NP0321BOX). After electrophoresis, the proteins were transferred onto a 0.45 μM PVDF transfer membrane (Immobilon-P, Millipore, #IPVH 00010) that was then blotted overnight with the appropriate primary antibodies at 4 °C: Actin (1:10,000; Sigma #A5441) or all the following antibodies from Cell Signalling Technology, phospho-MEK (1:1000; #9121), MEK (1:2000; #9126), phospho-P38 (1:1000; #4511), P38 (1:2000; #9218), phospho-JNK (1:1000; #4668), JNK (1:1000; #9258), phospho-AKT (1:500; #9271) and AKT (1:1000; #9272). Appropriate peroxidase-conjugated secondary antibodies (Jackson ImmunoResearch Laboratories #115-035-045 or ##115-035-003), were used to detect specific binding of primary antibodies and were visualized by enhanced chemiluminescence detection (Western Lightning Plus-ECL, PerkinElmer, #NEL105001EA). Image J software^110^ was used for densitometry analysis of immunoblots.

### RT-qPCR from PDX tumors

The soft (n = 6) and stiff (n = 6) OV26 PDX tumors removed from mice were snap-frozen in liquid nitrogen and store at − 80 °C. As previously published by the “Stress and Cancer” lab headed by Dr. F. Mechta-Grigoriou^44^, miRNeasy kit (Qiagen, #217004) was used to extract RNA according to the Manufacturer’s instructions. RNA concentrations were determined using a Nanodrop (Nanodrop Technologies). After reverse transcription of 1 μg of total RNA using the iScript Reverse Transcription Kit (Bio-Rad, #1708840), qPCR was performed using Power SYBR Green PCR Master Mix (Applied Biosystems, #4367659) on a Chromo4 Real-Time PCR detection System (Bio-rad) with primer concentration at 300 nM. All the primers were designed using the primerQuest tool (IDT). Limiting dilutions of primer were performed to assess their efficiency and we only used those with 95 to 100% efficacy. Data were analyzed using an Opticon Monitor (Bio-Rad) and normalized to Cyclophilin A mRNA. The primer sequences used were as follow (forward and reverse): CYR61: 5′-GAGTGGGTCTGTGACGAGGAT-3′; 5′-GGTTGTATAGGATGCGAGGCT-3′. CTGF: 5′-AGGAGTGGGTGTGTGACGA-3′; 5′-CCAGGCAGTTGGCTCTAATC-3′. ANXA3 5′-GAACCGAATAATGGTGTCCAG-3′; 5′-GTATGAGAAGAAGTAAGGTGGA-3′. ANKRD1 5′-AGTAGAGGAACTGGTCACTGG-3′; 5′-TGGGCTAGAAGTGTCTTCAGAT-3′.

### Microarrays

In order to classify HGSOC PDX models, including the models OV26, OV21 and OV33 used in our study, as Mesenchymal or Non-Mesenchymal, transcriptomic data from 13 PDX were combined with transcriptomic data from Curie cohort. As the two datasets were coming from two different experiments and two different platforms (Affymetrix Human Genome U133 Plus 2.0 array for patient tumors and Affymetrix Human Exon 1.0 ST array for PDX tumors), standardization per array was applied (for each gene, mean equal to zero and standard deviation equal to 1) in order to avoid technical artifacts. A hierarchical clustering using Euclidean distance and Complete agglomeration method was performed on 36 genes, correlated and anti-correlated with the miR-200a, defining Mesenchymal and Non-Mesenchymal tumors. Classification of PDX tumors was done by cutting the dendrogram in two parts. This method allowed discriminating between Mesenchymal and Non-Mesenchymal tumors.

We also performed microarrays to dissect the stiffness-associated pathways in the Mesenchymal PDX model OV26. The samples included 3 soft tumors, 5 peripheral regions of stiff tumors and 5 central regions of stiff tumors. PDX tumors removed from mice were snap-frozen in liquid nitrogen and store at − 80 °C. As soft tumors are of little size with homogeneous stiffness, only the center was dissected and analyzed by microarray. For each stiff tumor, we microdissected a peripheral fragment and a central fragment of similar size and weight (around 50 mg). RNA extraction was performed using the miRNeasy extraction kit (Qiagen, #217004). RNA concentration was determined using a Nanodrop spectrophotometer (A260/A280 ratio > 1.6; Nanodrop Technologies). After evaluating RNA quality using the Agilent RNA 6000 LabChip (Agilent RNA 6000 Nano Kit, Agilent, #5067-1511) electrophoresis test (integrity median value: 8.9), 100 ng of total RNA were hybridized in parallel on Human HTA 2.0 and Mouse MTA 1.0 microarrays (Affymetrix), in order to get the stromal and the epithelial gene expression profile of each sample. Normalization and gene-level expression measurements were computed using the iterPLIER algorithm. In order to verify the specie-specificity of the probesets, 4 “control” samples were also hybridized on both human and mouse microarrays. These “control” samples correspond to one human epithelial ovarian cell line (OV56), one mouse fibroblastic cell line (MRC5), one Universal Human Reference RNA and one Universal Mouse Reference RNA (Agilent technologies). The data were then transformed as Log2 + 1. Differential gene expression analyses were performed using an unpaired t-test, except when comparing the center and the periphery of each stiff tumor, where we used a paired-t-test. The differentially expressed genes showing a *P* value ≤ 0.05 and a fold change > 1.2 were kept and gene ontology enrichment analysis was then performed using the DAVID bioinformatics resources (https://david.ncifcrf.gov).

### Statistical analysis

As previously published by the “Stress and Cancer” lab headed by Dr. F. Mechta-Grigoriou^34,41,44^, data shown are means ± SEM (unless otherwise specified) and indicated in each figure legend. The number of tumors analyzed or the number of independent experiments performed are specified in each figure legend, with at least 3 independent experiments, unless otherwise specified. The statistical test types used are in agreement with data distribution and are indicated in figure legend. First, normality was checked using the Shapiro–Wilk test and then parametric or non-parametric two-tailed tests were applied accordingly. Differences were considered to be statistically significant at values of *P* ≤ 0.05. Spearman’s correlation test was used to evaluate the correlation coefficient between two parameters. All statistical analyses were performed using Prism software.

## Supplementary Information

Supplementary Information.
