# Supplementary material for: Stiffness increases with myofibroblast content and collagen density in mesenchymal high grade serous ovarian cancer
Source: Sci Rep. 2021 Feb 18;11:4219. doi: 10.1038/s41598-021-83685-0 (PMC7892556; doi:10.1038/s41598-021-83685-0)
Supplement: Supplementary file 1 — Supplementary Information. [file 41598_2021_83685_MOESM1_ESM.pdf]

SUPPLEMENTARY INFORMATION

Stiffness increases with myofibroblast content and collagen density in mesenchymal high grade serous ovarian cancer

Virginie Mieulet[1,2*#], Camille Garnier[1,2*], Yann Kieffer[1,2], Thomas Guilbert[3], Fariba Nemati[4], Elisabetta Marangoni[4], Gilles Renault[5], Foucauld Chamming's[6], Anne Vincent-Salomon[7] and Fatima Mechta-Grigoriou.[1,2#]

* These authors participate equally to this work

[1] Stress and Cancer Laboratory, Institut Curie, PSL Research University, Equipe labelisée Ligue Nationale Contre le Cancer, 26, rue d'Ulm, 75248 Paris Cedex 05, France

[2] Inserm, U830, Paris, F-75248, France

[3] IMAG'IC Facility, Institut Cochin, INSERM U1016, CNRS UMR 8104, Université de Paris UMR-S1016, F-75014 Paris, France

[4] Laboratory of Preclinical Investigation, Institut Curie, 26, rue d'Ulm, 75248 Paris Cedex 05, France

[5] Life Imaging Facility, University of Paris, "Imageries du Vivant" (PIV) platform, Institut Cochin, 22 rue Mechain, 75014 Paris, France

[6] Assistance publique-hôpitaux de Paris, HU Paris Ouest Site Georges Pompidou, 20 rue Leblanc, 75015 Paris, France

[7] Department of Diagnostic and Theranostic Medicine, Institut Curie Hospital Group, 26, rue d'Ulm, 75248 Paris Cedex 05, France

Running title: Stiffness during ovarian cancer growth

Address for correspondence: Dr Fatima Mechta-Grigoriou (ORCID Number: 0000-0002-3751-6989) and Dr. Virginie Mieulet (ORCID Number: 0000-0001-5104-639X). Email addresses: fatima.mechta-grigoriou@curie.fr and virginie.mieulet@curie.fr

Supplementary Figure 1. Supersonic Shear Wave Elastography is appropriate for measuring tumor stiffness, *in vivo* in HGSOC PDX model. (**A**) Correlation plot between mean tumor stiffness values obtained with Penetration mode and Resolution mode (n=22). Each point represents the mean of 2 to 4 stiffness maps for one Mesenchymal OV26 tumor at one time point. Correlation coefficient σ and *P* value are based on Spearman's rank correlation test. (**B**) Representative stiffness maps (SWE, top panel) and corresponding B-mode view (bottom panel) acquired with either Penetration or Resolution mode of the ultrafast imaging device (Aixplorer), in the transverse plan of one Mesenchymal OV26 tumor *in vivo*. (**C**) Representative view of Aixplorer sensitivity in a Mesenchymal OV26 tumor in contact with a new emerging nodule. The picture (bottom panel) illustrates the close contact between primary tumor and tiny new nodule emerging from the tumor. Dotted lines in the B-mode image (middle panel) define primary tumor and nodule sizes. The area and diameter are respectively 23 mm^2 and 5.2mm for the nodule or 87mm^2 and 13.7mm for the primary tumor. Stiffness map (SWE, top panel) shows the different pixel stiffness means between the nodule (24kPa) and the primary tumor (88kPa). All measurements were performed *in vivo* on anesthetized mice. (**D**) Representative stiffness maps (top panels) with the associated B-mode view (bottom panels) of one soft (left) and one stiff (right) Mesenchymal OV26 tumor with (+) or without (-) skin above the tumor implant. In the "- skin" conditions, the skin above the tumor has been cut and pushed aside allowing direct gel deposition on the tumor, in anesthetized mice. (**E**) Bar plot showing the mean tumor stiffness (n=3 stiffness maps) from the same conditions as in (H). Data are shown as mean ± SEM, *P* value is based on Student's t-test, ns stands for not significant. (**F**) Stiffness map (top panel) and associated B-mode view (bottom panel) of one Mesenchymal OV26 tumor partially covered by a gel layer injected between the skin and the tumor. The three layers are indicated on the B-mode image (white arrows). On SWE image, red circles identify either low skin stiffness in contact with the gel (left side, 5kPa) or high skin stiffness in contact with the tumor (right side, 70kPa). (**G**) Correlation plot between mean tumor area and mean tumor volume in Mesenchymal OV26 tumors (n=22). Each dot refers to a single tumor measurement at a given time (m=73). Correlation coefficient σ and *P* value are based on

Spearman's rank correlation test. (**H**) B-mode views in the transverse plan of representative Mesenchymal (OV26 and OV21), and Non-Mesenchymal (OV33) HGSOC over time, corresponding to the representative-colored stiffness maps shown in Fig. 1C. (A) stands for tumor area. t0 corresponds to the first day of tumor stiffness measurement, and the following days of measures are indicated. (**I**) Mean tumor area curves over time for Mesenchymal (OV26: n=20; OV21: n=22) (F) and Non-Mesenchymal (n=16) (G) PDX models. *P* values from Welch's t-test.

Supplementary Figure 2. Tumor stiffness-associated histological and molecular features. (**A**) Representative views of Ki67 (proliferation marker, 5x magnification) immunostaining in soft *versus* stiff Mesenchymal OV26 HGSOC. (**B**) Scatter plot of Ki67 histological score (Hscore) in the epithelial compartment (Hscore = staining intensity (0–4) × % of Ki67 positive epithelial cells) between soft (n=9) and stiff (n=11) Mesenchymal OV26 tumors. Data are shown as mean ± S.E.M. *P* value based on Mann Whitney test. (**C**) Representative views of HES staining showing necrotic areas (pink) in soft *versus* stiff Mesenchymal OV26 HGSOC. The necrotic areas have been delimited by hand, with green lines. Scale bar, 100µm. (**D**) Scatter plot showing the percentage of necrotic areas in soft (n=9) *versus* stiff (n=11) Mesenchymal OV26 tumors. Data are shown as mean ± S.E.M. *P* value based on Mann Whitney test. (**E**) Representative western blots showing the phosphorylated form (P-) and the total protein levels of MEK, P38, AKT, JNK-1 and JNK-2 in soft (n=10) and stiff (n=9) Mesenchymal OV21 tumors. Dashed line is used to delineate different parts from the same gel. Actin is used as an internal control for protein loading.

Supplementary Figure 3. Full western blots shown in main Figure 4.

(**A**) The western blots are from two different gels performed at the same time. The same tank was used for electrophoresis as well as for transfer. They were both blotted at the same time, in the same box, with the same primary or secondary antibody dilutions. They were then revealed at the same time, with the same time of exposure. Portions of these full membranes

1 and 2 (bottom), indicated with numbers, and showing the phosphorylated form (P-) and the total protein levels of AKT, JNK-1 and JNK-2 and Actin are shown in **Fig. 4D** (top), with the corresponding numbers. (**B**) Portions of these full western blots indicated by squares (left) and showing the phosphorylated form (P-) and the total protein levels of MEK, and Actin are presented in **Fig. 4H** (right).

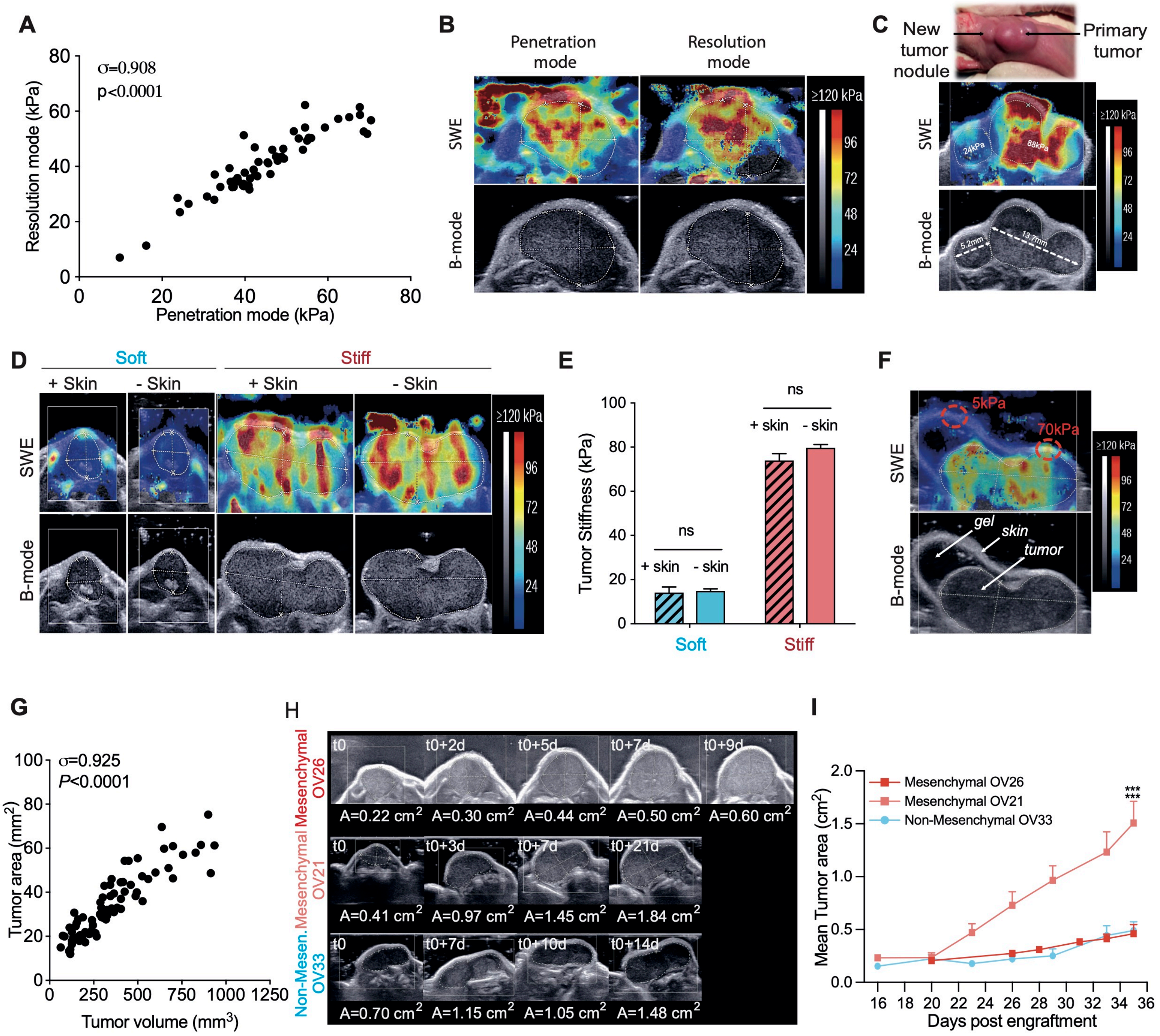
A
σ=0.908
p<0.0001
Resolution mode (kPa)
Penetration mode (kPa)
B
Penetration mode
Resolution mode
SWE
B-mode
≥120 kPa
C
New tumor nodule
Primary tumor
24kPa
88kPa
5.2mm
13.7mm
D
Soft
Stiff
+ Skin
- Skin
E
Tumor Stiffness (kPa)
ns
+ skin
- skin
Soft
Stiff
F
5kPa
70kPa
gel
skin
tumor
G
σ=0.925
P<0.0001
Tumor area (mm2)
Tumor volume (mm3)
H
Mesenchymal OV26
Mesenchymal OV21
Non-Mesen. OV33
t0
t0+2d
t0+5d
t0+7d
t0+9d
A=0.22 cm2
A=0.30 cm2
A=0.44 cm2
A=0.50 cm2
A=0.60 cm2
t0+3d
t0+21d
A=0.41 cm2
A=0.97 cm2
A=1.45 cm2
A=1.84 cm2
t0+10d
t0+14d
A=0.70 cm2
A=1.15 cm2
A=1.05 cm2
A=1.48 cm2
I
Mesenchymal OV26
Mesenchymal OV21
Non-Mesenchymal OV33
Mean Tumor area (cm2)
Days post engraftment
***
***

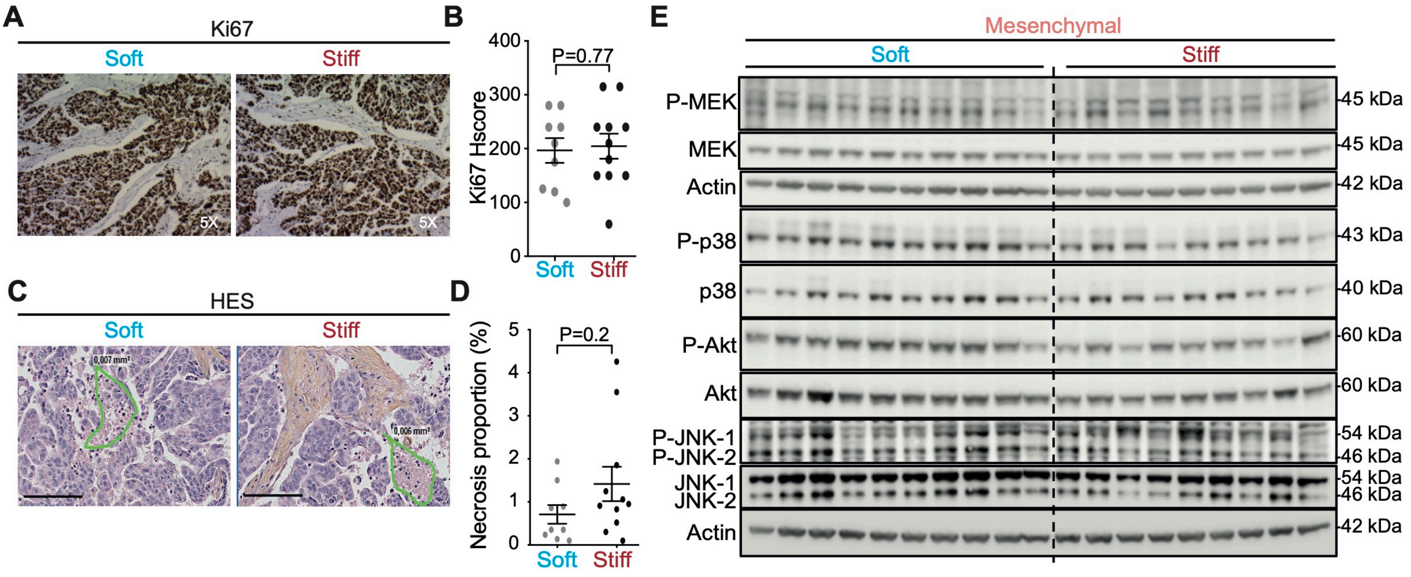
A
Ki67
Soft
Stiff
5X
5X
B
Ki67 Hscore
400
300
200
100
0
P=0.77
Soft
Stiff
C
HES
Soft
Stiff
D
Necrosis proportion (%)
5
4
3
2
1
0
P=0.2
Soft
Stiff
E
Mesenchymal
Soft
Stiff
P-MEK
MEK
Actin
P-p38
p38
P-Akt
Akt
P-JNK-1
P-JNK-2
JNK-1
JNK-2
Actin
45 kDa
45 kDa
42 kDa
43 kDa
40 kDa
60 kDa
60 kDa
54 kDa
46 kDa
54 kDa
46 kDa
42 kDa

A

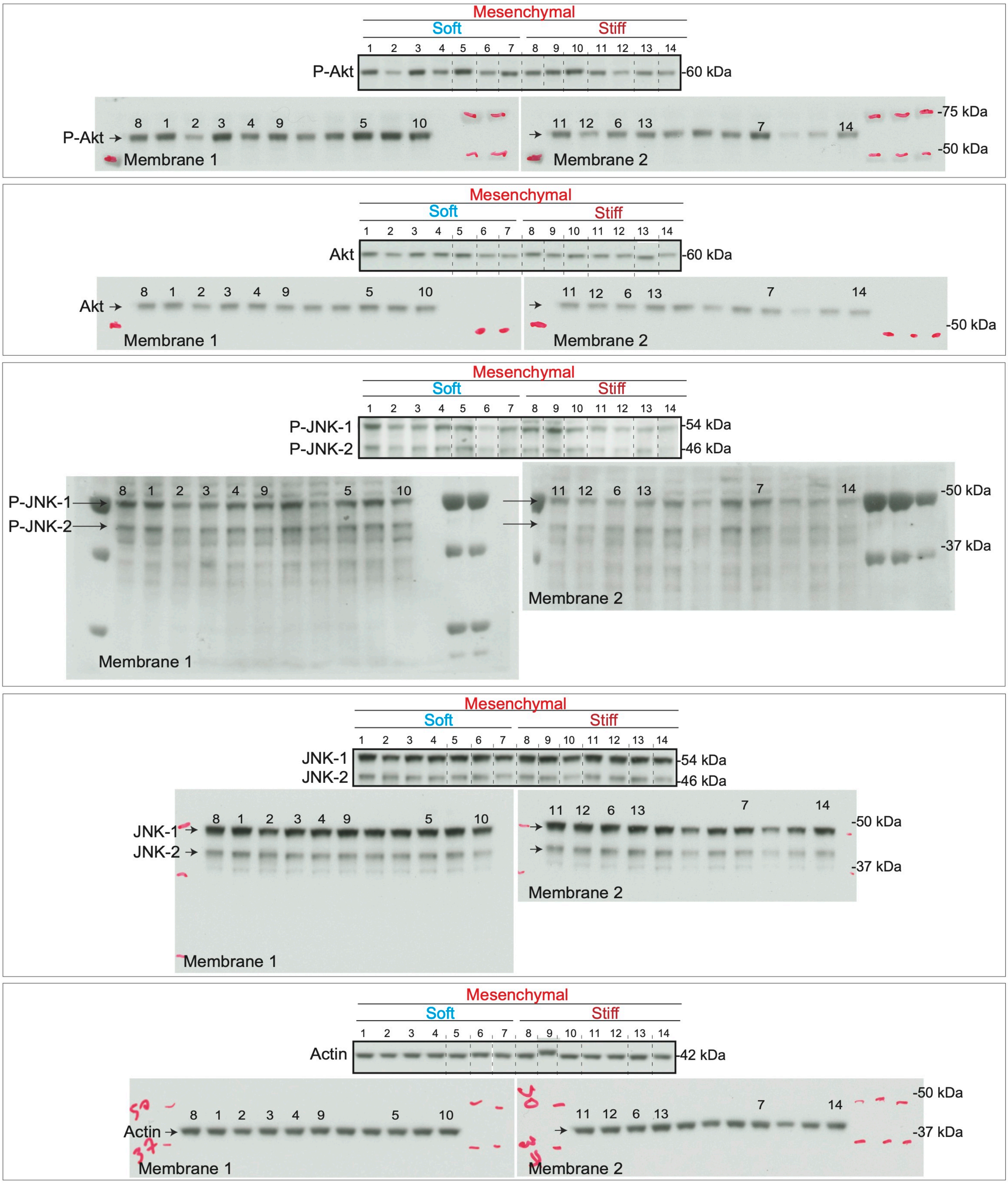
Mesenchymal
Soft
Stiff
1 2 3 4 5 6 7 8 9 10 11 12 13 14
P-Akt
-60 kDa
P-Akt
8 1 2 3 4 9 5 10
Membrane 1
11 12 6 13 7 14
Membrane 2
-75 kDa
-50 kDa
Akt
-60 kDa
Akt
-50 kDa
P-JNK-1
P-JNK-2
-54 kDa
-46 kDa
-37 kDa
JNK-1
JNK-2
Actin
-42 kDa

B

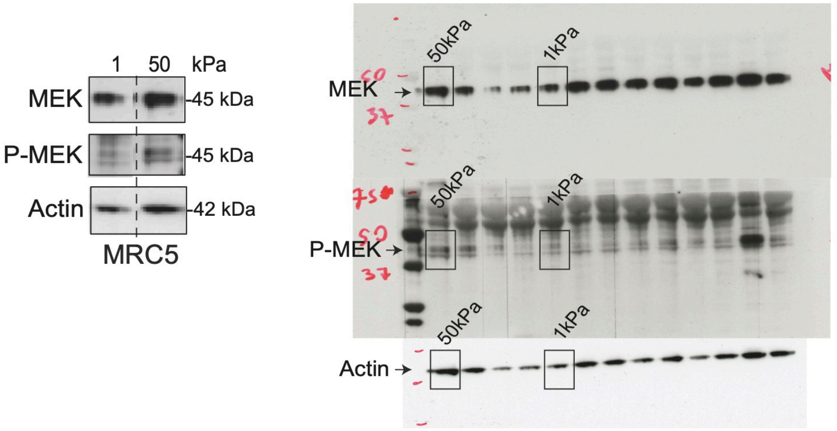
1 50 kPa
MEK
-45 kDa
P-MEK
-45 kDa
Actin
-42 kDa
MRC5
50kPa
1kPa
MEK
P-MEK
Actin
